# The existence and evolution of morphotypes in *Anolis* lizards: coexistence patterns, not adaptive radiations, distinguish mainland and island faunas

**DOI:** 10.7717/peerj.6040

**Published:** 2019-01-03

**Authors:** Steven Poe, Christopher G. Anderson

**Affiliations:** Department of Biology and Museum of Southwestern Biology, University of New Mexico, Albuquerque, NM, USA

**Keywords:** Ecomorphs, Community ecology, Sympatry, Assembly rules, Lizards, Evolution, Adaptive radiation

## Abstract

The evolution of distinct ecologies and correlated morphologies (“ecomorphs,” in combination) among similar species allows sympatric occupation of diverse microhabitats. Particular ecomorphs may evolve repeatedly, that is, convergently, as separate lineages arrive at similar solutions. Caribbean *Anolis* lizards (anoles) are a classic ecomorph system, particularly well-studied for the diverse morphotypes resulting from adaptive radiations. But few studies have analyzed the equally species-diverse mainland *Anolis*. Here, we use clustering analyses of nine traits for 336 species of *Anolis* to objectively identify morphological groups (morphotypes). We analyze the presence of recovered morphotypes on mainland and islands in general and relative to the composition of 76 mainland and 91 island anole assemblages. We test for evolutionary convergence of morphotypes within and between mainland and island environments by mapping our recovered morphotypes onto recent phylogenetic estimates and by analyzing four of our measured traits using program SURFACE. We find that particular morphotypes tend to be restricted to either mainland or island environments. Morphotype diversity and convergence are not concentrated within either island or mainland environments. Morphotype content of assemblages differs between mainland and island areas, with island assemblages displaying greater numbers of morphotypes than mainland assemblages. Taken with recent research, these results suggest a restructuring of one of the classic adaptive radiation stories and a reconsideration of research concerning island–mainland faunal differences. Island radiations of anoles are unexceptional relative to mainland radiations with regard to species count, rates of speciation and phenotypic evolution, morphotype diversity, and rates of convergence. But local island assemblage appear to be more diverse than mainland assemblages. The explanation for this assemblage disparity may reside in one of the classic hypothesized island–mainland environmental differences (i.e., greater numbers of predators/competitors/environmental complexity on the mainland). Similarity between mainland and island anole radiations may indicate exceptional evolution in the anole clade overall or ordinary evolution in an extraordinarily studied clade.

## Introduction

Similar species coexist by occupying different ecological niches (Hardin, 1960). Occupation of such niches often results in the evolution of associated morphologies (Darwin, 1859), for example aquatic species may have fins even if they evolved from terrestrial forms, and species that live in caves may be blind and colorless but descended from functionally eyed, colorful ancestors. Subtler examples of morphological correlations with ecology such as larger lizards living higher in trees and short-tailed lizards tending to occupy twigs led to the concept of the ecomorph in Greater Antillean *Anolis* lizards (Rand & Williams, 1969; Williams, 1972). In the Caribbean *Anolis* system, similar ecomorphological types evolved repeatedly (i.e., convergently; Losos et al., 1998) and in some cases, such as Jamaica and Cuba, via independent intraisland adaptive radiations (Losos, 2009; see relationships in Poe et al., 2017). The ecomorph concept has since been applied to diverse systems such as spiders (Gillespie, 2004), finches (McKay & Zink, 2014), and cichlids (Malinsky et al., 2015).

Most detailed studies of ecomorphs have analyzed island faunas, with the implicit or explicit corollary that mainland clades are less given to ecomorphological divergence (Pinto et al., 2008). Mainland environments are thought to be older and more complex than islands, with greater numbers of predators and competitors and a greater diversity of habitats (MacArthur & Wilson, 1967; Carlquist, 1974; Schluter, 1988). These factors may suppress ecomorphological divergence among close relatives, whereas the comparatively open niche space of islands may encourage adaptive radiation producing multiple ecomorphological types (Schluter, 2000). However, few studies have quantitatively compared mainland and island ecomorphs (but see Schaad & Poe, 2010; Moreno-Arias & Calderon-Espinosa, 2016), so these suppositions remain to be tested on a large scale.

Most studies of “ecomorphs” do not include ecological data but rather measure morphology and assume a link between morphology and ecology. For example, the classic paper establishing evolutionary convergence of ecomorphs in *Anolis* (Losos et al., 1998) included morphological data for 46 species but no ecological data. Fortunately for the extendability of our morphology-based results, a strong ecology–morphology link has been established in *Anolis* (Collette, 1961; Rand & Williams, 1969; Williams, 1983; Losos, 1990; Calsbeek & Irschick, 2007). Thus although our study of anole morphology does not directly address nonmorphological aspects of assemblages, it is highly unlikely that our results are uninformative about ecology and ecomorphs. Still, in the interest of forthrightness we will generally refer to our analyzed units as “morphotypes” rather than ecomorphs.

Here, we use an unprecedented morphological dataset of 336 species of *Anolis* (174 mainland, 162 island), a new dataset of *Anolis* assemblage content (76 mainland, 91 island), a recently-published comprehensive phylogeny of *Anolis* (Poe et al., 2017), and approaches similar to Mahler et al. (2013) and Moreno-Arias & Calderon-Espinosa (2016) to ask the following questions: (1) Are particular morphotypes generally restricted to mainland or island environments, or are they present across these regions? Niche conservatism (Wiens et al., 2010) would lead to geographic restriction of morphotypes, but mainland–island convergence and frequent dispersal between mainland and island regions would cause broader distribution of morphotypes. (2) Are morphotypes more diverse on islands relative to the mainland, or vice-versa? The known ecomorphological diversity in the Greater Antilles (Williams, 1972; Losos, 2009) suggests an expectation of greater numbers of morphotypes on islands. (3) Do island or mainland assemblages contain greater numbers of morphotypes per assemblage? Some Greater Antillean anole assemblages are famously ecomorphologically diverse (e.g., Soroa, Cuba; Losos et al., 2003; Rodríguez Schettino et al., 2010), which suggests an expectation of greater morphotype diversity within island assemblages generally. (4) Has convergence of morphotypes occurred on the mainland, as has been shown on the islands (Losos et al., 1998)? (5) Is convergence of morphotypes more frequent within island and mainland environments than between these environments? If island and/or mainland environments are more similar within vs. between environments, then natural selection may concentrate convergence within these regions. We interpret our results in terms of recent work on adaptive radiation and coexistence, generally and in mainland and island *Anolis*.

## Materials and Methods

### Data

We collected nine morphological characters from one to 15 specimens of 336 species of *Anolis* (a recent study (Armstead & Poe, 2015) suggests *N* = 1 is an adequate sample size for our purposes). These data are accessible in the Supplemental Information (Table S1). Body size (snout to vent length; SVL) was measured from tip of snout to anterior edge of cloaca; head length (HDL) from tip of snout to anterior edge of ear; tail length (TAL) from anterior edge of cloaca to tip of tail; toe length (TOL) from base of fourth toe to tip, including claw; femur length (FML) from ventral longitudinal midline laterally to knee. Dorsal (DSC) and ventral (VSC) scale size was measured as number of scales counted longitudinally in 5% of SVL. Number of toe lamellae (LAM) was counted using Williams et al.’s (1995) method. Size of head scales (HSC) was measured as number of scales across the snout at the level of the second canthal scales.

Most of these traits have theoretical or demonstrated functional utility for lizards. For example, head length may be related to feeding habits (Verwaijen, Van Damme & Herrel, 2002), limb length correlates with microhabitat use (Irschick, 2002), scale size is related to dessication rate (Soulé & Kerfoot, 1966), and body size affects many if not most life history traits (Peters, 1983). Some of our analyzed traits are strongly correlated with body size so we collected these traits (HDL, TAL, TOL, FML) in units of SVL. Although there is no single correct way to scale measurements based on overall size (see Packard & Boardman, 1988), many authors use residuals from linear regression on body size instead of our approach of simply dividing by body length. However, for our data, our approach gives results that are nearly completely correlated with body size residuals (e.g., shown in Fig. S1 of Poe & Latella, in press). Therefore we adopt our approach for practical reasons (e.g., it is not necessary to perform a new regression every time new data are added).

Some of our studied traits are not normally distributed (in particular, LAM and HSC are skewed with long right tails), and we found that ln-transformations allowed better separation of groups in our clustering analyses (see below). Therefore we ln-transformed all traits before analyses.

We compiled *Anolis* species composition for 76 island and 91 mainland assemblages from throughout the range of *Anolis*. We included assemblages of at least two species for which either (1) we had “ground-truthed” species content (i.e., by field work from 2003 to 2015 conducted by SP’s lab), or (2) species content had been assessed by repeated, reliable surveys (Powell & Henderson, 2012). Because we were concerned about pseudoreplication (Hurlbert, 1984), we stipulated that each analyzed assemblage include a unique combination of species. Assemblage information is available in the Supplemental Information (Table S2). Field work was approved by the University of New Mexico Institutional Animal Care and Use Committee (protocol 16-200554-MC).

For evolutionary analyses (see below), we used the maximum clade credibility (MCC) tree of Poe et al. (2017) and a 100-tree sample from Poe et al.’s (2017) Bayesian postburnin sample, both pruned to include only the 336 species scored for morphological traits.

#### Analyses

We analyzed the existence and evolution of morphotypes in mainland and island *Anolis* using two distinct analytical approaches: clustering analyses and likelihood-based reconstruction of morphotypes.

In order to objectively identify groups of morphologically similar species using our nine traits of morphology, we employed average-linkage clustering analysis of Euclidean distances for our nine ln-transformed traits in Stata (StataCorp, 2011). We identified sets of clusters based on a stopping rule incorporating Je(2)/Je(1) values and pseudo-T-squared values (Duda, Hart & Stork, 2001). In particular, we identified sets of groups that satisfied both Je(2)/Je(1) > 0.9 and pseudo-T-squared < 2.0. Groups identified in this Duda–Hart analysis were used in additional analyses. We recognize that any grouping cutoff is necessarily arbitrary, but note first that this approach should be preferable to initial attempts at recognizing morphotypes (ecomorphs) that were either nonquantitative (Rand & Williams, 1969) or identified groups prior to quantitative treatment (Losos et al., 1998), and note second that we have assessed our results across four plausible groupings (see below), which should give some credence to conclusions that are consistent across groupings.

We first tallied the number of exclusively mainland, exclusively island, and shared island–mainland morphotypes according to each set of groups that satisfied our Je(2)/Je(1) and pseudo-T-squared criteria (see below). We determined the significance of each value according to null tests where mainland/island occupancy was considered random across morphotypes according to current frequency; probabilities of each outcome were calculated based on this distribution.

We calculated the number of species per morphotype for each assemblage under each set of morphotypes that satisfied our Je(2)/Je(1) and pseudo-T-squared criteria. This statistic is a measure of the morphotype diversity within an assemblage, scaled for the number of species in the assemblage (among our sampled assemblages, mainland assemblages are slightly more species-diverse than island assemblages; ${\bar x_{{\rm{mainland}}}} = 4.6$ species/assemblage, ${\bar x_{{\rm{island}}}} = 3.8$ species/assemblage, *P* = 0.007, Mann–Whitney *U*-test). We compared this statistic between mainland and island assemblages using a Mann–Whitney *U*-test (the distributions of this statistic are strongly non-normal—when large numbers of morphotypes are recognized (see below), many values are minimal, i.e., 1.0).

We examined the evolutionary convergence of morphotypes using two approaches. First, we performed SURFACE (Ingram & Mahler, 2013) analyses on estimated trees from Poe et al. (2017), pruned of species not scored for our morphological traits (i.e., trees of 336 taxa). We analyzed four traits—body length, relative FML, relative HDL, and scales across the snout—rather than all nine because the authors of SURFACE recommend using two to four traits for best results (Ingram & Mahler, 2013). Among our scored traits, we use these four because we judge them to be supported by the most evidence suggesting functional importance for lizards (See Introduction). The forward phases of the SURFACE analysis was conducted with default priors. For the backward phase in SURFACE, we specified the starting model as the final Hansen model returned in the forward phase and applied default priors. Alternatively, we could have specified a minimum acceptable model improvement (ΔAIC_c_ per iteration) or maximum number of steps. However, we opted to allow SURFACE to choose the best model at each step, accept any improvement, and continue iterating until improvement to the model ceased. We performed analyses on the MCC tree of Poe et al. (2017) and seven additional trees from their MrBayes posterior sample (a greater number of trees was not evaluated due to computational constraints—one forward/backward SURFACE run for these data on a single tree takes over 2 weeks on computers at the University of New Mexico Center for Advanced Research Computing). We reconstructed geography (mainland vs. island) on trees using likelihood in Mesquite (Maddison & Maddison, 2017) and tallied “regime changes” (i.e., changes in morphotype) occurring in mainland and island branches.

As an alternative to the SURFACE analyses, we mapped the largest sample of morphotypes recovered in the clustering analysis onto 100 trees from Poe et al.’s (2017) postburnin sample. We reconstructed mainland and island branches using likelihood in Mesquite, and reconstructed morphotype changes using parsimony. We counted the number (range) of singular and convergent evolutions of morphotypes, and whether changes were reconstructed to occur in mainland or island branches.

## Results

Four morphotype groupings satisfied our Duda–Hart criteria: *N* = 8, 18, 27, 73 groups (i.e., morphotypes) among the 336 included species. Table S3 in the Supplemental Information shows morphotype assignment by species. Table 1 shows the distribution of morphotypes across mainland and island environments. The number of exclusively mainland and exclusively island morphotypes is significantly high for some morphotype groupings, and the number of morphotypes shared across island and mainland and island environments is significantly low. That is, particular morphotypes are concentrated within mainland and island environments. Island areas do not harbor a significant preponderance of morphotypes relative to mainland areas; the number of morphotypes in each region is approximately equal under each set of morphotypes (Table 1).

**Table 1:** Morphotypes are concentrated within island and mainland environments.

Number of morphotypes	Number of exclusively mainland morphotypes	Number of exclusively island morphotypes	Number of morphotypes found on both mainland and islands
8	1 (0.8; *P* = 0.64)	2 (0.8; *P* = 0.14)	5 (6.4)
18	4 (1.9; *P* = 0.06)	5 (1.8; *P* < 0.01)	9 (14.3)
27	6 (2.9; *P* < 0.05)	5 (2.7; *P* = 0.06)	16 (21.4)
73	22 (12.1; *P* < 0.01)	23 (11.2; *P* < 0.01)	28 (49.7)

**Note:**

Neither mainland nor island environments harbor greater numbers of morphotypes. Expected values and probability of at least as many morphotypes under random assignment of morphotypes to mainland and island environments are in parentheses.

Table 2 shows that island assemblages are composed of proportionately greater numbers of morphotypes than mainland assemblages for all groupings.

**Table 2:** Island assemblages include more morphotypes per assemblage than mainland assemblages.

Number of morphotypes	Number of species per morphotype per assemblage, islands (Mean, standard deviation)	Number of species per morphotype per assemblage, mainland (Mean, standard deviation)	*P*-value, Mann–Whitney test
8	1.59 (0.63)	3.21 (1.59)	<0.0001
18	1.20 (0.33)	1.79 (0.71)	<0.0001
27	1.14 (0.30)	1.36 (0.36)	<0.0001
73	1.06 (0.14)	1.18 (0.22)	<0.0001

**Figure 1:**
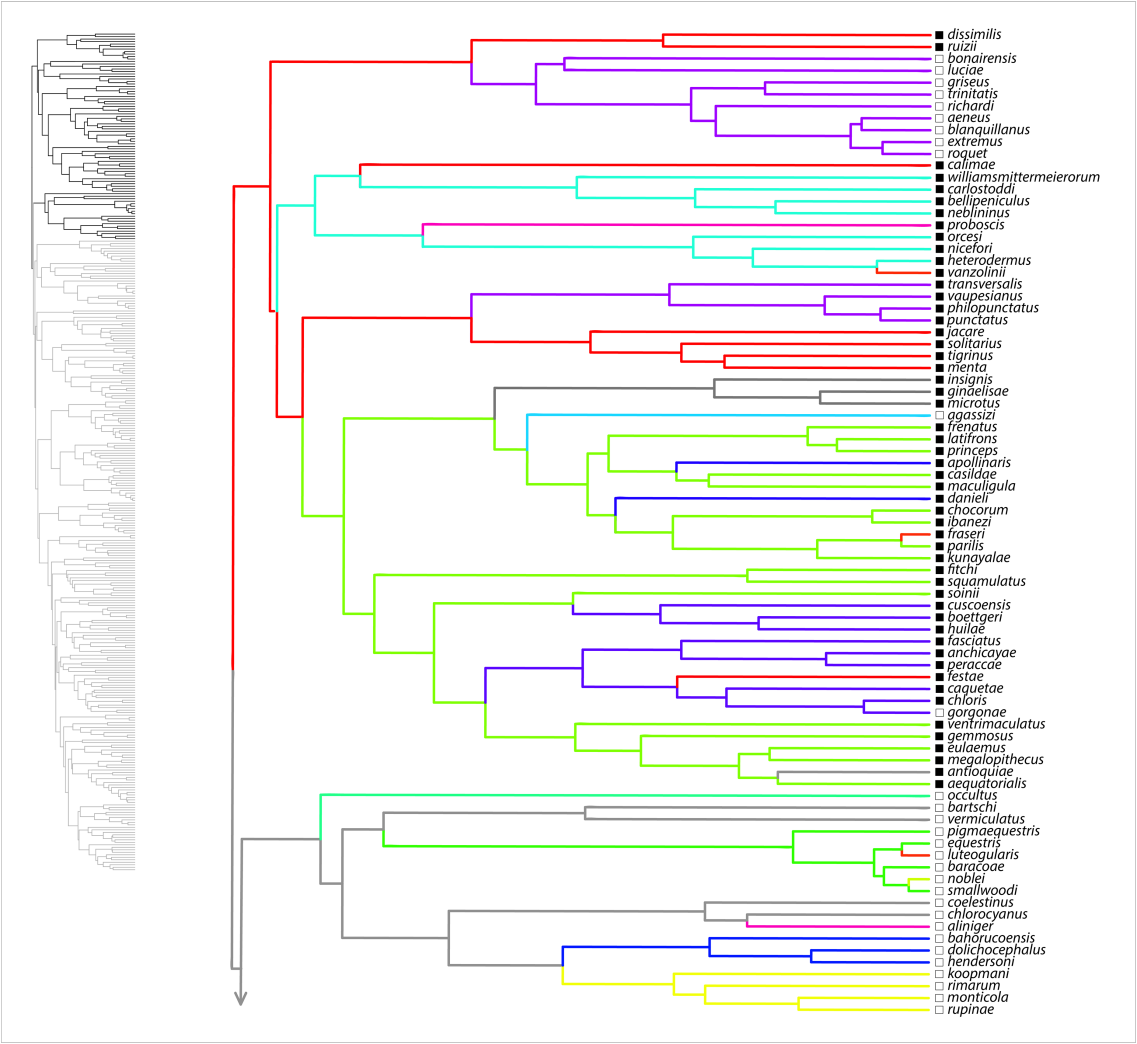
SURFACE results on maximum clade credibility tree. Lineages are colored according to estimated evolved regimes. Filled squares are mainland species, open squares are island species.

**Figure 2:**
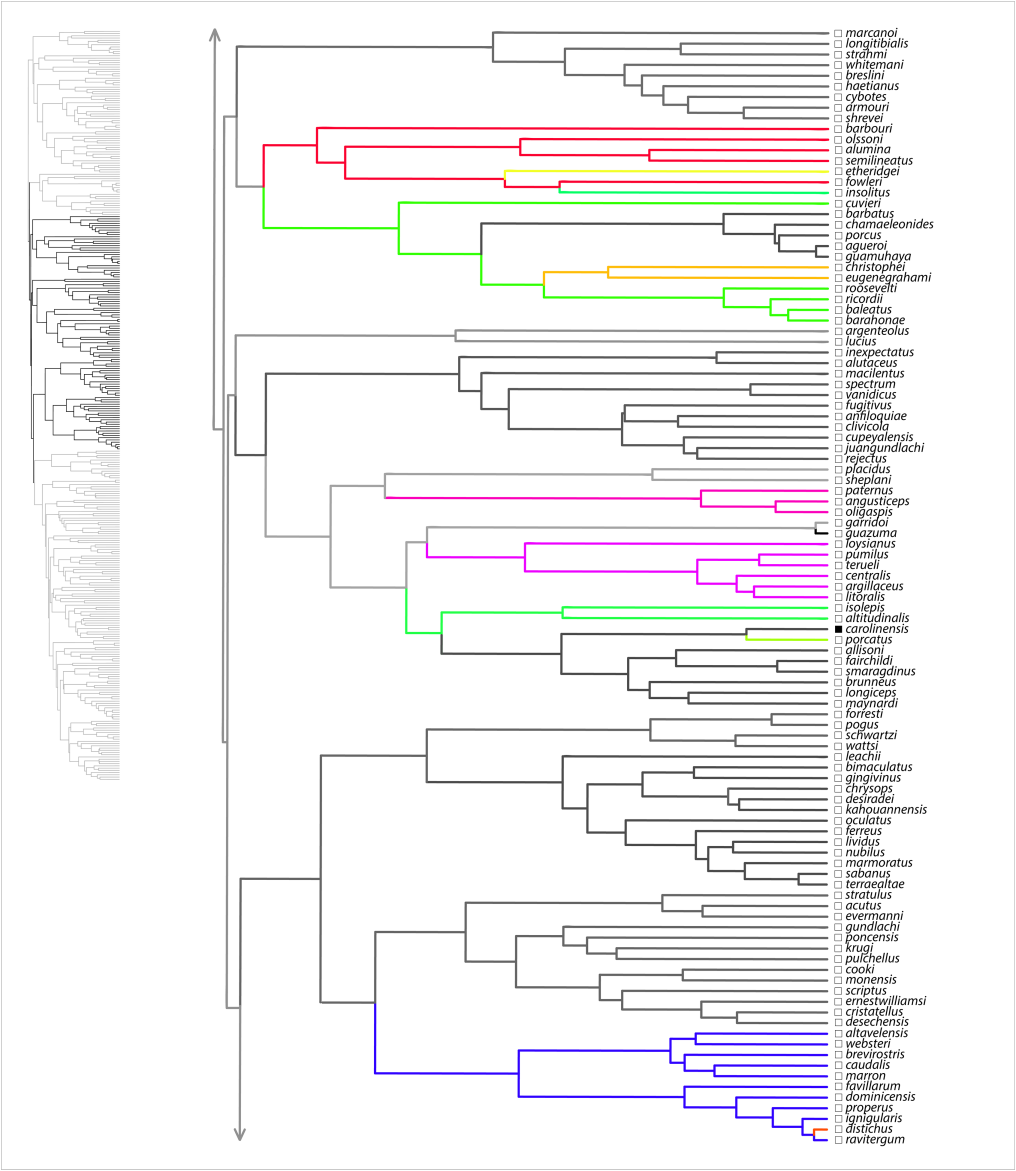
SURFACE results on maximum clade credibility tree for *Anolis* clades *Ctenonotus*, *Ctenocercus Audantia*, *Schmidtanolis*, *Xiphosurus*. Lineages are colored according to estimated evolved regimes. Filled squares are mainland species, open squares are island species.

**Figure 3:**
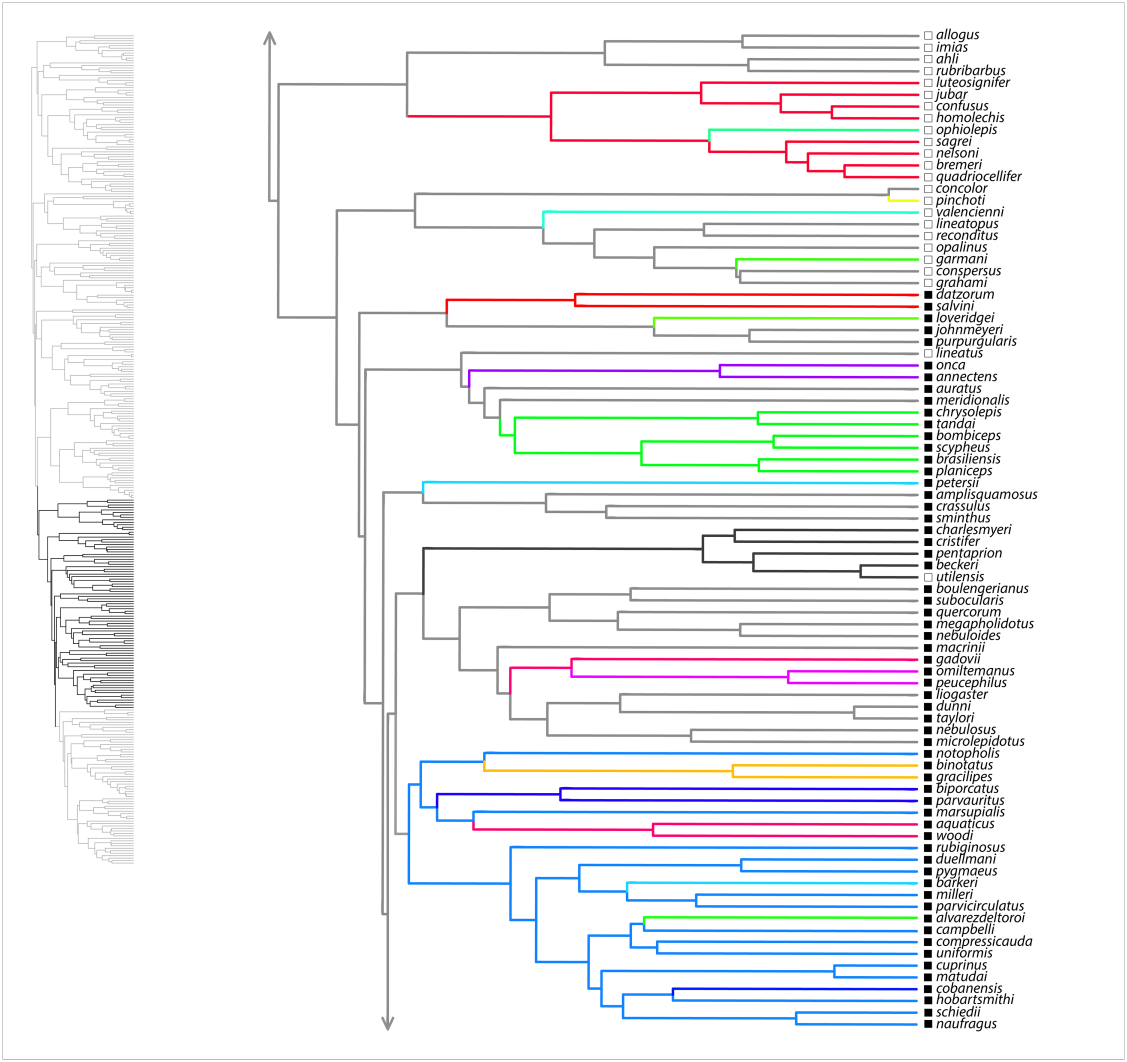
SURFACE results on maximum clade credibility tree for *Anolis* clades *Trachypilus*, *Placopsis*, part of *Draconura*. Lineages are colored according to estimated evolved regimes. Filled squares are mainland species, open squares are island species.

**Figure 4:**
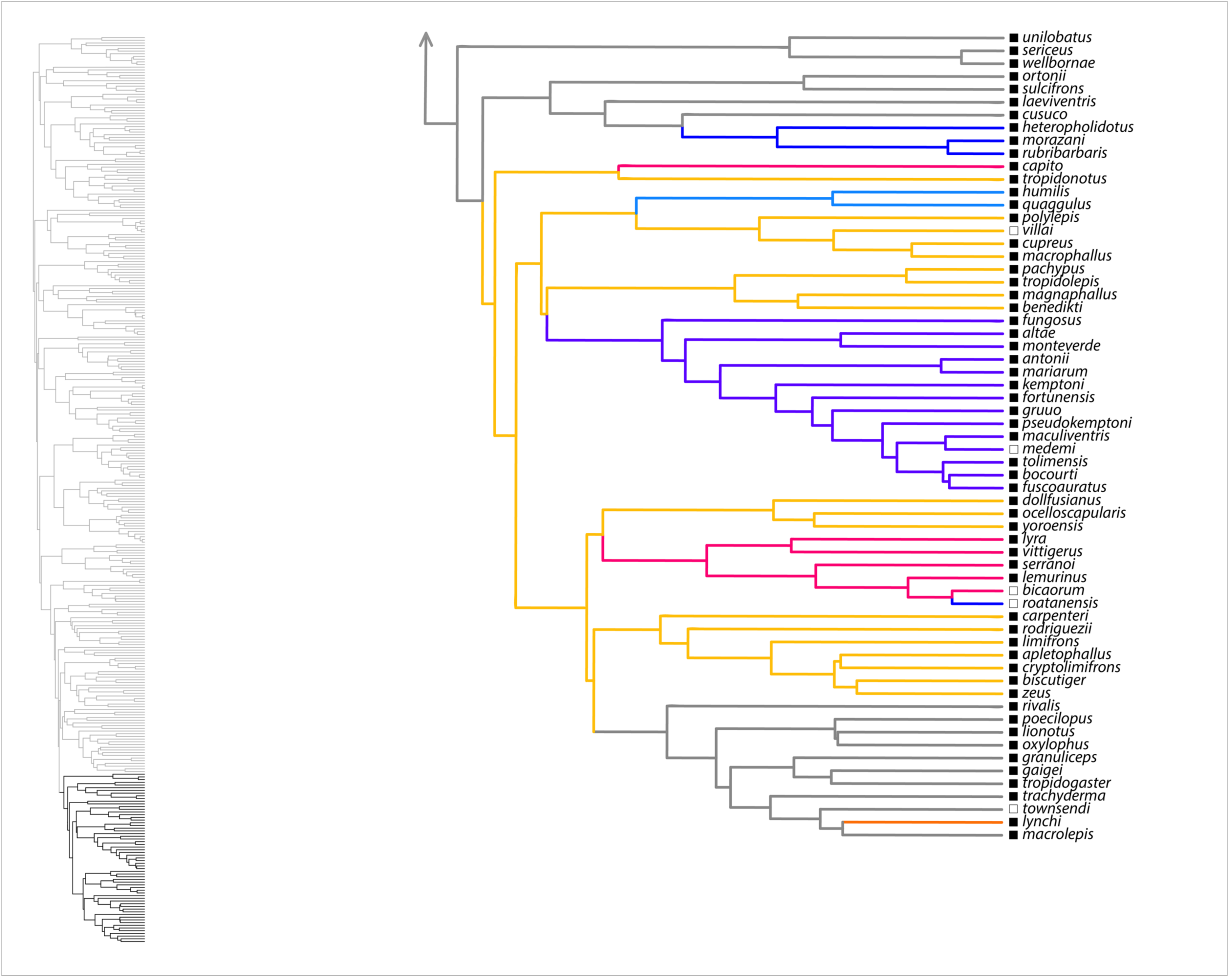
SURFACE results on maximum clade credibility tree for part of *Anolis* clade *Draconura*. Lineages are colored according to estimated evolved regimes. Filled squares are mainland species, open squares are island species.

Here, we summarize results from the SURFACE analysis on the MCC tree, with the range of values among eight analyzed trees noted in parentheses. Our SURFACE analysis produced model improvements (ΔAIC_c_) of 416.1 (368.1–480.4) and 305.2 (153.7–305.2) in forward and backward phases, respectively. The final Hansen model included 74 (49–74) regime shifts toward 37 (25–38) adaptive regimes. Of these, 60 (39–60) convergent shifts to 23 (15–23) shared adaptive regimes and 14 (10–17) nonconvergent shifts to adaptive regimes were identified. Among convergent regimes, we found six (three to six) instances of convergence within islands, six (three to seven) instances of convergence within the mainland, and 11 (6–14) instances of convergence between the mainland and islands. Of nonconvergent regimes, five (four to six) occur on the mainland and nine (seven to nine) occur on islands. Overall, there were 36 (28–36) regime shifts on islands and 38 (21–38) on the mainland. Figures 1–4 show the results of the SURFACE analysis on the MCC tree. Table 3 summarizes SURFACE results for the MCC tree and gives examples of estimated instances of convergence between species.

**Table 3:** Convergent regime changes estimated in SURFACE analysis.

Regime	No. changes within mainland	No. changes within islands	Example species
1	3	0	*Anolis calimae* (SA), *A. salvini* (CA)
2	2	1	*A. extremus* (SL), *A. punctatus* (SA)
3	1	1	*A. heterodermus* (SA), *A. valencienni* (JM)
4	1	2	*A. proboscis* (SA), *A. paternus* (CB)
5	2	1	*A. fraseri* (SA), *A. luteogularis* (CB)
6	2	1	*A. petersii* (CA), *A. barkeri* (CA)
7	2	0	*A. frenatus* (CA), *A. loveridgei* (CA)
8	3	0	*A. apollinaris* (SA), *A. biporcatus* (CA)
9	3	0	*A. soinii* (SA), *A. chloris* (SA)
10	0	3	*A. equestris* (CB), *A. cuvieri* (PR)
11	0	2	*A. noblei* (CB), *A. porcatus* (CB)
12	1	1	*A. bahorucoensis* (HS), *A. cobanensis* (CA)
13	0	3	*A. monticola* (HS), *A. pinchoti* (PD)
14	0	2	*A. sagrei* (CB), *A. fowleri* (HS)
15	0	2	*A. insolitus* (HS), *A. isolepis* (CB)
16	1	1	*A. loysianus* CB), *A. omiltemanus* (MX)
17	1	2	*A. brevirostris* (HS), *A. roatanensis* (RT)
18	1	1	*A. distichus* (HS), *A. lynchi* (SA)
19	0	2	*A. occultus* (PR), *A. ophiolepis* (CB)
20	2	1	*A. alvarezdeltoroi* (MX), *A. scypheus* (SA)
21	4	0	*A. gadovii* (MX), *A. aquaticus* (CA)
22	2	0	*A. notopholis* (SA), *A. humilis* (CA)
23	2	1	*A. gracilipes* (SA), *A. polylepis* (CA)

**Note:**

Native areas are in parentheses: SA, South America; CA, Central America; SL, Southern Lesser Antilles; JM, Jamaica; HS, Hispaniola; RT, Roatan; MX, Mexico; CB, Cuba; PR, Puerto Rico; PD, Isla Providencia.

**Figure 5:**
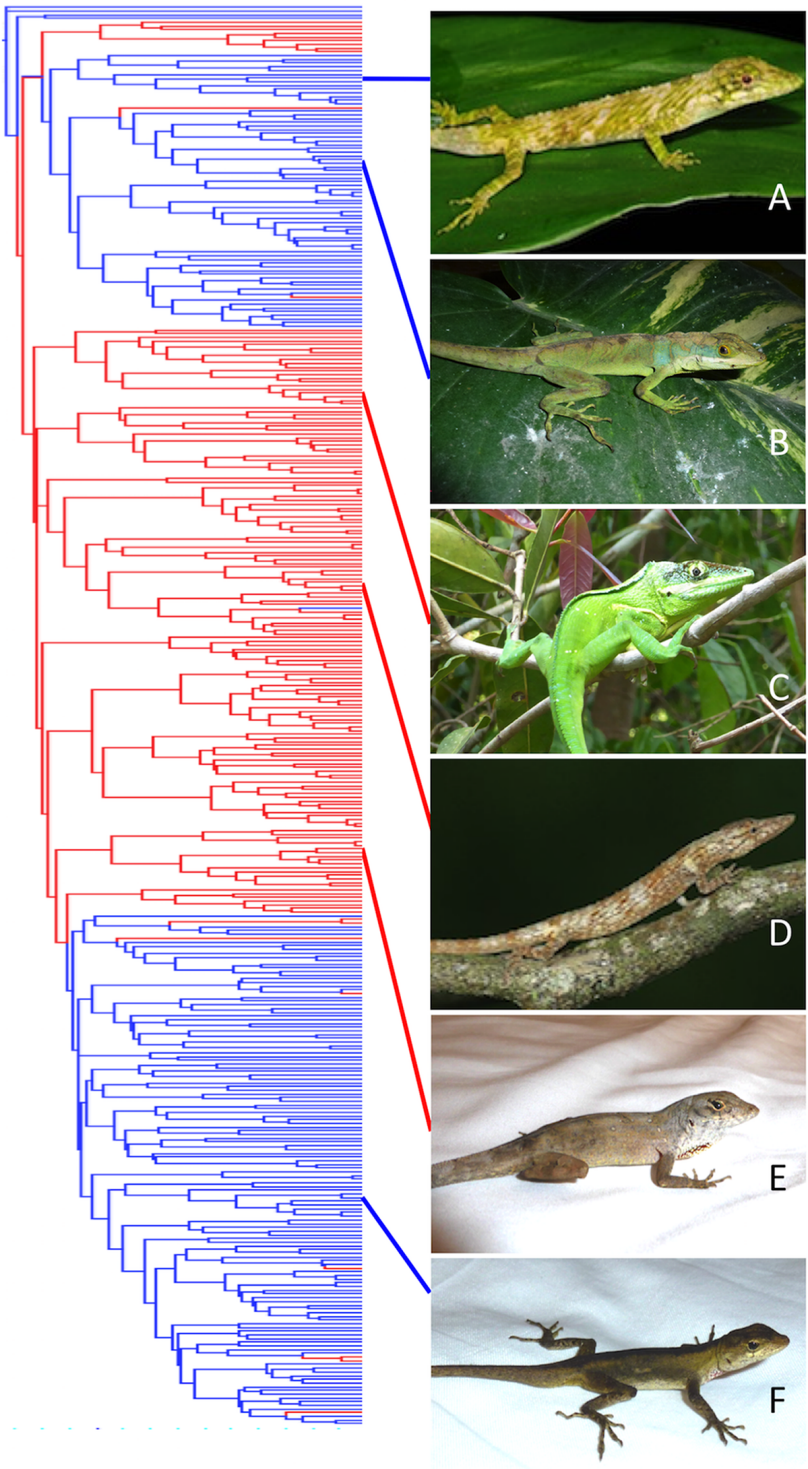
Phylogenetic distribution of mainland and island habitation in *Anolis* and three potential examples of mainland–island convergence. Island lineages in red, mainland in blue according to parsimony reconstruction on one of Poe et al.’s (2017) postburnin MCMC trees. Species names removed for clarity. Species are (A) *Anolis orcesi* (Ecuador; Photo by Tom Kennedy), (B) *A. apollinaris* (Colombia; Photo by Steven Poe), (C) *A. luteogularis* (Cuba; Photo by Steven Poe), (D) *A. sheplani* (Dominican Republic; Photo by Alejandro Sánchez Muñoz), (E) *A. sagrei* (Cuba; Photo by Steven Poe), (F) *A. boulengerianus* (Mexico; Photo by Steven Poe). Species pairs that represent potential cases of mainland–island convergence according to our clustering analyses are *A. orcesi* and *A. sheplani*, *A. apollinaris* and *A. luteogularis*, *A. sagrei* and *A. boulengerianus*.

Table S4 of the Supplemental Information summarizes results of the parsimony analyses reconstructing changes in the 73-morphotype dataset across 100 postburnin trees. A total of 11–14 morphotypes was reconstructed to evolve nonconvergently on islands and 9–13 on the mainland. A total of 9–12 morphotypes was estimated to evolve convergently within islands and 11–15 within the mainland. A total of 26 morphotypes was estimated to evolve convergently between mainland and island environments. Overall, 105–123 changes between morphotypes were reconstructed to occur on islands and 116–141 to occur on the mainland. Figure 5 shows mainland-island reconstruction and three examples of likely convergence of morphotypes on the MCC tree.

## Discussion

Mainland and island environments have been hypothesized to present differing selection pressures on faunas that may result in alternative coexistence patterns, rates of evolution, amounts of convergence, and levels of ecomorphological diversity. We found most of these aspects to be similar across mainland and island environments among our tested characters in a well-studied tropical lizard clade, the *Anolis*. These results may be interpreted as compatible with a “null hypothesis” of no environmental effect on our studied traits; indicative of character, methodology, and/or taxon choice that failed to capture actual island or mainland effects; or suggestive of the broader distinctiveness of the anole clade or the neotropical region where anoles reside. Below we expand on some of these points and discuss some future prospects for studies of adaptive radiation and mainland–island comparisons.

### Existence and coexistence of morphotypes

We have endeavored here to examine patterns of objectively identifiable *Anolis* morphotypes with regard to geography and evolution. Many of our recovered morphotype groupings make sense according to previous phenetic or gestalt-based inferences regarding anole groups. For example, group 8 of our 8-morphotype set includes only the two large phenacosaur species (*heterodermus, vanzolinii*); group 7 of our 18-morphotype set is composed of mainland and island “twig” *Anolis* (island: *angusticeps, oligaspis, garridoi, guazuma, paternus, placidus, sheplani, insolitus;* mainland*: fungosus, orcesi*); group 17 in our 27-morphotype set includes the traditionally recognized Caribbean “Crown Giant” species (*baleatus, baracoae, barahonae, equestris, noblei, pigmaequestris, ricordii, roosevelti, smallwoodi, cuvieri, garmani, luteogularis);* and group 71 of our 73-morphotype set includes only the distinctive *Chamaeleolis* clade *Anolis (agueroi, barbatus, chamaeleonides, guamuhaya, porcus*). Although there certainly are exceptions to the intuitively satisfying exemplars listed above (mainly overlumped groups in our 8-morphotype set, oversplit groups in our 73-morphotype set), we believe the recovered groups are at least as reasonable as previous categorizations (e.g., ecomorphs, Williams, 1972; ecomodes, Nicholson et al.’s 2012), and have the advantage of being objectively and quantitatively identified (see also Mahler et al., 2013; Moreno-Arias & Calderon-Espinosa, 2016). Further, we attempted to address the sensitivity of some of our conclusions by testing our inferences based on a stopping rule that resulted in four sets of potential morphotype groupings.

The finding that morphotypes are significantly restricted within mainland and island environments (Table 1) should not be surprising, for biogeographic regions. If species tend to evolve from similar, nearby species—that is, if there is some niche and morphological conservatism during evolution (Wiens et al., 2010)—then similar morphotypes would be likely to be found within a region. This pattern could dissolve if dispersal between mainland and island environments occurred frequently and/or morphotype convergence between mainland and island environments was common. But neither dispersal (Poe et al., 2017) nor mainland–island convergence (see below) appears common enough to override biogeographic inertia in *Anolis*.

The expectation that a greater number of morphotypes would be found on islands was not fulfilled. Numbers of morphotypes were roughly comparable between mainland and island regions (Table 1). This result may be surprising given that the anoles of the Greater Antilles are literally a textbook example of adaptive radiation producing great ecomorphological diversity (e.g., Freeman, Quillan & Allison, 2013: Chapter 24). Our finding contributes to the growing body of work suggesting that the mainland anole radiation may rival the island clades in adaptive radiation characteristics—a result that is compatible with both exceptional evolution in both areas and unexceptional evolution within the anole clade (see below). Poe et al. (2018) found no increase in speciation rate in island *Anolis*, and no increase in rates of morphological evolution in island forms if outgroup nonanoles were taken into account. If selective regimes differ between island and mainland environments, these differences are not manifest in the existence of greater numbers of morphotypes among island *Anolis*.

However, although the overall number of morphotypes is approximately equal between islands and the mainland (Table 1), there appears to be a structural difference within island assemblages relative to mainland assemblages (Table 2). That is, island assemblages are composed of greater numbers of morphotypes, a result that might be expected given the established ecomorphological diversity in the Greater Antilles (Williams, 1983; Losos, 2009) and the demonstrated levels of within-assemblage morphological variability found by Anderson & Poe (2018). The result of greater morphotype diversity within assemblages on islands may be attributable to local selective forces of extra-*Anolis* competition and predation being strong enough on the mainland to constrain *Anolis* assemblage variability. Niche space for anoles in local mainland assemblages (i.e., small, arboreal insectivores) may be filled by corytophanid lizards, *Avicularia* spiders, or plethodontid salamanders, none of which are found in the Greater Antilles. Meanwhile in island environments the paucity of nonanole competitors and predators allows freer coexistence of multiple anole morphotypes.

These traditional explanations for mainland–island faunal differences of increased complexity, longer history, and greater numbers of predators and competitors on the mainland (Schluter, 1988), suffice as initial potential hypotheses to explain mainland-island assemblage differences in *Anolis* (Table 2). We also note the existence of multiple diverse “duplicate” assemblages on the islands (e.g., the Soroa and Sierra de Trinidad assemblages on Cuba are ecomorphologically similar, as are many two-species assemblages in the Lesser Antilles), which might indicate early evolution of ecomorphs followed by vicariant speciation or ecomorph assortment. But these explanations leave open the explanation for the surprising juxtaposition of these differences with the comparable overall number of morphotypes on mainland and island environments (Table 1), and the apparent similarity of mainland and island anole radiations (Poe et al., 2018). Perhaps adaptive radiation proceeds similarly in mainland and island environments, with similar morphotypes produced, but local coexistence is still dictated by the traditionally hypothesized mainland–island differences discussed above. Teasing apart the causal aspects of such scenarios is likely to be difficult.

### Evolution and convergence of morphotypes

Our evolutionary analyses demonstrated copious convergence of morphotypes within and between island and mainland environments (Table 3; Figs. 1–4; Table S4 of Supplemental Information). In the SURFACE analysis of the MCC tree, the distribution of morphotype convergence (six mainland, six island, 11 mainland/island) nearly exactly reflects the expected distribution of convergence under an equiprobable distribution of convergence events. For example, given two evolutions of each “regime” (i.e., one instance of convergence; actual mean evolutions per regime is 2.6) and equal numbers of mainland and island branches (we analyzed 174 mainland, 162 island species), the binomial expectation for a random distribution of *N* = 23 instances of convergence would be 5.75 mainland, 5.75 island, and 11.5 occurring in both mainland and island. Thus, these data show no tendency for convergence to be concentrated within both or either island and/or mainland environments. These results, which are reinforced in the parsimony morphotype analyses (Table S4 of the Supplemental Information), suggest that mainland environments produce comparable convergence relative to island environments. Although the SURFACE analyses incorporate a phylogenetic estimate that includes weakly supported nodes deep in the tree (see Poe et al., 2017), we have confidence that our broad conclusion of comparable island–mainland convergence is robust to tree uncertainty, for two reasons. First, we incorporated some uncertainty by analyzing multiple trees in both our SURFACE and parsimony morphotype studies. Second, most cases of convergence occur between members of strongly supported clades (Fig. 1). In summary, the island environment does not appear to be especially conducive for adaptive radiation of morphotypes (ecomorphs); mainland radiations converge just as do island radiations, at similar rates.

### Evolution of *Anolis* lizard diversity on islands and mainland

Recent work has shown that (1) mainland and island radiations of *Anolis* do not differ in speciation rate (Poe et al., 2018), (2) mainland and island radiations of *Anolis* do not differ in rates of morphological evolution, unless outgroup effects are ignored (Poe et al., 2018), (3) *Anolis* ecomorphs evolved convergently in the Greater Antilles (Losos et al., 1998; Mahler et al., 2013), (4) island assemblages tend to display greater morphological diversity than mainland assemblages (Anderson & Poe, 2018; this paper), (5) comparable numbers of morphotypes occur on mainland and islands (this paper), and (6) rates of convergence of morphotypes are similar within and between mainland and islands (this paper). So what is to be made of these patterns?

Greater Antillean *Anolis* are one of the classic adaptive radiations, producing ecomorphological diversity whilst exploiting new, open niches (Williams, 1972). However, it appears that the only currently established measured difference between mainland and island *Anolis* radiations involves local assemblage content, with island assemblages being more diverse (Anderson & Poe, 2018; this paper). And even this result is tenuous, as it rests on our selection of assemblages for study (we submit that there can be no truly “random” sampling of assemblages, and that the concerns of Hurlbert (1984) are equally true today for community ecology). The result of mostly unexceptional island evolution may warrant a reconsideration of the textbook *Anolis* narrative, where Greater Antillean anole clades have been advanced as archetypal adaptive radiations (Freeman, Quillan & Allison, 2013), and perhaps of the adaptive radiation concept in general, which has focused on island cases, at least in its rigorous operational forms.

Regarding *Anolis*, this result of island-mainland similarity does not necessarily force a new paradigm, but rather, potentially, an expansion of it. We emphasize that our results do not contradict or diminish the importance of the wealth of knowledge of adaptive radiation, ecomorphology, and coexistence gleaned from studying island anoles. But perhaps *Anolis* itself, rather than Greater Antillean *Anolis*, is the exceptional case of evolutionary radiation. Although this shift was anticipated by some *Anolis* biologists (E. Williams, 1992, personal communication; Losos, 2009), its embracement should not proceed uncritically. In particular, it must be realized that focusing on lineage comparisons is inappropriate without associating those lineages with reconstructed hypothesized causal evolutionary factors. Put more bluntly, asking whether *Anolis* (or any other clade) is exceptional is not a biologically interesting question unless one articulates what it is about anoles that might make them exceptional. The dewlap (but see Poe et al., 2018), toe lamellae, scales, phenotypic plasticity, habitat use, the Greater Antilles, or mainland cordilleras could all be tested as causal triggers for *Anolis* diversity. But to be valid these tests must be conducted with comparison to appropriate “null” squamate lineages and including pertinent nonanole lineages such as geckos with toepads and *Draco*s with dewlaps.

## Conclusions

Here, we have used a large, nearly comprehensive dataset to identify groups of morphologically similar species of *Anolis* lizards. We compared the existence, evolution, and assemblage content of these morphotypes in mainland and island environments. We found that morphotypes are concentrated within mainland and island environments, probably due to biogeographic reasons, but morphotype convergence is not unusually common within either region. Island assemblages include greater proportions of morphotypes, possibly due to a dearth of predators and competitors in island environments. Interpreted in light of recent research, these results suggest a reconsideration of *Anolis* as one of the classic island adaptive radiations. There currently is little evidence that rates of trait evolution, speciation, or convergence are elevated in island relative to mainland *Anolis*, and overall species and morphological diversity appear similar between these regions. Explaining the differences in assemblage structure of mainland and island *Anolis* may be a focus of future work.

## Supplemental Information

10.7717/peerj.6040/supp-1 Supplemental Information 1 Table S1. Morphological data for species of *Anolis* analyzed here. Entries are raw and ln-transformed mean values for traits for each species. Traits are body length (SVLMAX, lnsvl), relative femur length (FML/SVL, lnfml), relative head length (HL/SVL, lnhl), relative toe length (TOL/SVL, lntol), relative tail length (TAL/SVL, lntal), number of ventral scales in 5% of body length (V5%SVL, lnv5), number of dorsal scales in 5% of body length (D5%SVL, lnd5), number of toe lamella (LM, lnlm), number of scales across the snout at the level of the second canthal scales (HS, lnshs).

10.7717/peerj.6040/supp-2 Supplemental Information 2 Table S2. Assemblages analyzed here.

10.7717/peerj.6040/supp-3 Supplemental Information 3 Table S3. Morphotypes assigned according to the clustering approach and stopping rules discussed in the text.

10.7717/peerj.6040/supp-4 Supplemental Information 4 Table S4. Number of evolutionary changes to each morphotype reconstructed to occur in island or mainland environments under 73-morphotype grouping and parsimony criterion. Ranges (e.g., “1–2”) represent variation across 100 phylogenetic estimates. Numbers refer to morphotypes listed in Table S3 of the Supplemental Information.

## Additional Information and Declarations

### Data Availability

The following information was supplied regarding data availability: The raw data are provided in the Supplemental Tables.

## References

[ref-1] Anderson CG, Poe S (2018). Phylogeny, biogeography and island effect drive differential evolutionary signals in mainland and island lizard assemblages. Zoological Journal of the Linnaean Society.

[ref-2] Armstead J, Poe S (2015). Use of an exemplar versus use of a sample for calculating summary metrics of morphological traits in comparative studies of *Anolis* lizards. Herpetological Review.

[ref-43] Calsbeek R, Irschick DJ (2007). The quick and the dead: correlational selection on morphology, performance, and habitat use in island lizards. Evolution.

[ref-3] Carlquist S (1974). Island biology.

[ref-41] Collette S (1961). Correlations between ecology and morphology in anoline lizards from Havana, Cuba and southern Florida. Bulletin of the Museum of Comparative Zoology.

[ref-4] Darwin C (1859). On the origin of species by means of natural selection, or, the preservation of favoured races in the struggle for life.

[ref-5] Duda RO, Hart PE, Stork DG (2001). Pattern classification.

[ref-6] Freeman S, Quillan K, Allison L (2013). Biological science.

[ref-7] Gillespie RG (2004). Community assembly through adaptive radiation in Hawaiian spiders. Science.

[ref-8] Hardin G (1960). The competitive exclusion principle. Science.

[ref-9] Hurlbert SH (1984). Pseudoreplication and the design of ecological field experiments. Ecological Monographs.

[ref-10] Ingram T, Mahler DL (2013). SURFACE: detecting convergent evolution from comparative data by fitting Ornstein-Uhlenbeck models with stepwise Akaike Information criterion. Methods in Ecology and Evolution.

[ref-11] Irschick DJ (2002). Evolutionary approaches for studying functional morphology: examples from studies of performance capacity. Integrative and Comparative Biology.

[ref-42] Losos JB (1990). The Evolution of form and function: morphology and locomotor performance in West Indian lizards. Evolution.

[ref-12] Losos JB (2009). Lizards in an evolutionary tree: ecology and adaptive radiation of anoles.

[ref-13] Losos JB, Jackman TR, Larson A, De Queiroz K, Rodriguez-Schettino L (1998). Contingency and determinism in replicated adaptive radiations of island lizards. Science.

[ref-14] Losos JB, Leal M, Glor RE, De Queiroz K, Hertz PE, Rodríguez Schettino L, Lara AC, Jackman TR, Larson A (2003). Niche lability in the evolution of a Caribbean lizard community. Nature.

[ref-15] MacArthur RH, Wilson EO (1967). The theory of Island biogeography.

[ref-16] Maddison WP, Maddison DR (2017). Mesquite: a modular system for evolutionary analysis. 3.2. http://mesquiteproject.org.

[ref-17] Mahler DL, Ingram T, Revell LJ, Losos JB (2013). Exceptional convergence on the macroevolutionary landscape in island lizard radiations. Science.

[ref-18] Malinsky M, Challis RJ, Tyers AM, Schiffels S, Yohey T, Ngatunga BP, Misk EA, Durbin R, Genner MJ, Turner GF (2015). Genomic islands of speciation separate cichlid ecomorphs in an East African crater lake. Science.

[ref-19] McKay BD, Zink RM (2014). Sisyphean evolution in Darwin’s finches. Biological Reviews of the Cambridge Philosophical Society.

[ref-20] Moreno-Arias RA, Calderon-Espinosa ML (2016). Patterns of morphological diversification of mainland *Anolis* lizards from northwestern South America. Zoological Journal of the Linnean Society.

[ref-21] Nicholson KE, Crother BI, Guyer C, Savage JM (2012). It is time for a new classification of anoles (Squamata: Dactyloidae). Zootaxa.

[ref-22] Packard GC, Boardman TJ (1988). The misuse of ratios, indices, and percentages in ecophysiological research. Physiological Zoology.

[ref-23] Peters RH (1983). The ecological implications of body size.

[ref-24] Pinto G, Mahler DL, Harmon LJ, Losos JB (2008). Testing the island effect in adaptive radiation: rates and patterns of morphological diversification in Caribbean and mainland *Anolis* lizards. Proceedings of the Royal Society of London B: Biological Sciences.

[ref-25] Poe S, Latella I (in press). Empirical test of the native-nonnative distinction: native and nonnative assemblages of *Anolis* lizards are similar in morphology and phylogeny. Functional Ecology.

[ref-26] Poe S, Nieto-Montes De Oca A, Torres-Carvajal O, De Queiroz K, Velasco JA, Truett B, Gray LN, Ryan MJ, Köhler G, Ayala-Varela F, Latella I (2017). A phylogenetic, biogeographic, and taxonomic study of all extant species of *Anolis* (Squamata; Iguanidae). Systematic Biology.

[ref-27] Poe S, Nieto-Montes De Oca A, Torres-Carvajal O, De Queiroz K, Velasco JA, Truett B, Gray LN, Ryan MJ, Köhler G, Ayala-Varela F, Latella I (2018). Comparative evolution of an archetypal adaptive radiation: innovation and opportunity in *Anolis* lizards. American Naturalist.

[ref-28] Powell R, Henderson RW (2012). Island lists of West Indian amphibians and reptiles. Bulletin of the Florida Museum of Natural History.

[ref-29] Rand AS, Williams EE (1969). The anoles of La Palma: aspects of their ecological relationships. Breviora.

[ref-30] Rodríguez Schettino L, Losos JB, Hertz PE, De Queiroz K, Chamizo AR, Leal M, González VR (2010). The Anoles of Soroa: aspects of their ecological relationships. Breviora.

[ref-31] Schaad EW, Poe S (2010). Patterns of ecomorphological convergence among mainland and island *Anolis* lizards. Biological Journal of the Linnean Society.

[ref-32] Schluter D (1988). The evolution of finch communities on islands and continents: Kenya vs. Galapagos. Ecological Monographs.

[ref-33] Schluter D (2000). The ecology of adaptive radiation.

[ref-34] Soulé M, Kerfoot C (1966). On the climatic determination of scale size in a lizard. Systematic Zoology.

[ref-35] StataCorp (2011). Stata Statistical Software: Release 12.

[ref-36] Verwaijen D, Van Damme R, Herrel A (2002). Relationships between head size, bite force, prey handling efficiency and diet in two sympatric lacertid lizard. Functional Ecology.

[ref-37] Wiens JJ, Ackerly DD, Allen AP, Anacker BL, Buckley LB, Cornell HV, Damschen EI, Davies TJ, Grytnes J-A, Harrison SP, Hawkins BA, Holt RD, McCain CM, Stephens PR (2010). Niche conservatism as an emerging principle in ecology and conservation biology. Ecology Letters.

[ref-38] Williams EE (1972). The origin of faunas. Evolution of lizard congeners in a complex island fauna: a trial analysis. Evolutionary Biology.

[ref-39] Williams EE, Huey RB, Pianka ER, Schoener TW (1983). Ecomorphs, faunas, island size, and diverse end points in island radiations of *Anolis*. Lizard Ecology: Studies of a Model Organism.

[ref-40] Williams EE, Rand H, Rand AS, O’Hara RJ (1995). A computer approach to the comparison and identification of species in difficult taxonomic groups. Breviora.

