# Supplementary material for: The existence and evolution of morphotypes in Anolis lizards: coexistence patterns, not adaptive radiations, distinguish mainland and island faunas
Source: PeerJ. 2019 Jan 3;6:e6040. doi: 10.7717/peerj.6040 (PMC6321754; doi:10.7717/peerj.6040)
Supplement: Supplemental Information 1 — Entries are raw and ln-transformed mean values for traits for each species. Traits are body length (SVLMAX, lnsvl), relative femur length (FML/SVL, lnfml), relative head length (HL/SVL, lnhl), relative toe length (TOL/SVL, lntol), relative tail length (TAL/SVL, lntal), number of ventral scales in 5% of body length (V5%SVL, lnv5), number of dorsal scales in 5% of body length (D5%SVL, lnd5), number of toe lamella (LM, lnlm), number of scales across the snout at the level of the second canthal scales (HS, lnshs). [file peerj-07-6040-s001.pdf]

Anolis species	mainland0 island1	SVLMAX	lnsvl	FML/SVL	lnfml	HDL/SVL	lnhdl	TOL/SVL	lntol
acutus	1	67	4.20	0.29	-1.23	0.30	-1.20	0.19	-1.66
aeneus	1	77	4.34	0.25	-1.38	0.26	-1.35	0.15	-1.90
aequatorialis	0	92	4.52	0.32	-1.13	0.24	-1.41	0.29	-1.24
agassizi	1	127	4.84	0.31	-1.18	0.25	-1.37	0.23	-1.47
agueroi	1	154	5.04	0.20	-1.61	0.29	-1.24	0.13	-2.04
ahli	1	59	4.08	0.30	-1.22	0.26	-1.33	0.22	-1.51
aliniger	1	60	4.09	0.23	-1.46	0.30	-1.22	0.17	-1.77
allisoni	1	91	4.51	0.23	-1.46	0.33	-1.11	0.14	-1.97
allogus	1	63	4.14	0.29	-1.25	0.27	-1.29	0.21	-1.56
altae	0	44	3.78	0.26	-1.36	0.26	-1.36	0.18	-1.71
altavelensis	1	52	3.95	0.29	-1.23	0.25	-1.38	0.17	-1.77
altitudinalis	1	52	3.95	0.22	-1.51	0.32	-1.15	0.13	-2.06
alumina	1	40	3.69	0.27	-1.31	0.28	-1.29	0.21	-1.56
alutaceus	1	38	3.64	0.26	-1.36	0.29	-1.25	0.17	-1.77
alvarezdeltoroi	0	69	4.23	0.37	-1.00	0.24	-1.42	0.21	-1.56
amplisquamosus	0	44	3.78	0.27	-1.30	0.26	-1.34	0.16	-1.83
anchicayae	0	63	4.14	0.27	-1.32	0.24	-1.44	0.21	-1.56
anfiloquiae	1	43	3.76	0.29	-1.24	0.28	-1.27	0.17	-1.77
angusticeps	1	52	3.95	0.20	-1.61	0.30	-1.20	0.13	-2.04
annectens	0	78	4.36	0.29	-1.24	0.25	-1.39	0.23	-1.47
antioquiae	0	79	4.37	0.24	-1.43	0.24	-1.43	0.24	-1.43
antonii	0	55	4.01	0.28	-1.28	0.25	-1.40	0.18	-1.71
apletophallus	0	47	3.85	0.33	-1.11	0.24	-1.41	0.22	-1.51
apollinaris	0	106	4.66	0.26	-1.36	0.29	-1.23	0.21	-1.56
aquaticus	0	73	4.29	0.32	-1.15	0.25	-1.38	0.19	-1.66
argenteolus	1	56	4.03	0.30	-1.21	0.27	-1.31	0.18	-1.71
argillaceus	1	48	3.87	0.22	-1.50	0.28	-1.28	0.17	-1.77

armouri	1	67	4.20	0.29	-1.23	0.31	-1.17	0.19	-1.66
auratus	0	54	3.99	0.24	-1.42	0.24	-1.41	0.17	-1.77
bahorucoensis	1	51	3.93	0.28	-1.26	0.32	-1.15	0.21	-1.56
baleatus	1	180	5.19	0.26	-1.34	0.29	-1.25	0.18	-1.71
baracoae	1	172	5.15	0.25	-1.39	0.30	-1.21	0.16	-1.83
barahonae	1	160	5.08	0.26	-1.34	0.28	-1.26	0.18	-1.71
barbatus	1	170	5.14	0.21	-1.54	0.31	-1.16	0.14	-1.97
barbouri	1	47	3.85	0.32	-1.15	0.27	-1.31	0.18	-1.71
barkeri	0	101	4.62	0.30	-1.20	0.23	-1.47	0.16	-1.83
bartschi	1	78	4.36	0.32	-1.13	0.28	-1.28	0.19	-1.66
beckeri	0	61	4.11	0.23	-1.47	0.27	-1.33	0.13	-2.04
bellipeniculus	0	70	4.25	0.23	-1.47	0.28	-1.27	0.15	-1.90
benedikti	0	49	3.89	0.34	-1.08	0.24	-1.43	0.21	-1.56
bicaorum	1	67	4.20	0.32	-1.13	0.25	-1.40	0.19	-1.66
bimaculatus	1	123	4.81	0.27	-1.30	0.28	-1.29	0.20	-1.61
binotatus	0	51	3.93	0.31	-1.16	0.25	-1.38	0.20	-1.61
biporcatus	0	98	4.58	0.26	-1.36	0.27	-1.30	0.18	-1.71
biscutiger	0	38	3.64	0.30	-1.20	0.26	-1.33	0.21	-1.56
blanquillanus	1	85	4.44	0.26	-1.35	0.24	-1.44	0.18	-1.71
bocourti	0	43	3.76	0.29	-1.24	0.24	-1.43	0.17	-1.77
boettgeri	0	69	4.23	0.26	-1.33	0.23	-1.47	0.22	-1.51
bombiceps	0	67	4.20	0.36	-1.02	0.24	-1.44	0.20	-1.61
bonairensis	1	71	4.26	0.27	-1.32	0.26	-1.33	0.19	-1.66
boulengerianus	0	63	4.14	0.32	-1.13	0.26	-1.35	0.17	-1.77
brasiliensis	0	66	4.19	0.32	-1.14	0.24	-1.44	0.22	-1.54
bremeri	1	72	4.28	0.27	-1.32	0.27	-1.30	0.20	-1.61
breslini	1	57	4.04	0.31	-1.17	0.32	-1.14	0.20	-1.60
brevirostris	1	53	3.97	0.29	-1.24	0.26	-1.34	0.15	-1.90
brunneus	1	76	4.33	0.24	-1.45	0.32	-1.14	0.14	-1.97

calimae	0	59	4.08	0.22	-1.53	0.26	-1.34	0.16	-1.83
campbelli	0	51	3.93	0.32	-1.14	0.28	-1.27	0.24	-1.43
capito	0	95	4.55	0.34	-1.08	0.25	-1.39	0.23	-1.47
caquetae	0	58	4.06	0.26	-1.35	0.27	-1.33	0.17	-1.77
carlostoddi	0	56	4.03	0.21	-1.56	0.26	-1.33	0.15	-1.90
carolinensis	0	71	4.26	0.24	-1.41	0.30	-1.20	0.17	-1.77
carpenteri	0	45	3.81	0.27	-1.31	0.24	-1.44	0.15	-1.90
casildae	0	104	4.64	0.31	-1.17	0.26	-1.33	0.24	-1.43
caudalis	1	53	3.97	0.26	-1.35	0.26	-1.35	0.17	-1.77
centralis	1	42	3.74	0.23	-1.49	0.27	-1.30	0.16	-1.83
chamaeleonides	1	177	5.18	0.22	-1.51	0.30	-1.22	0.13	-2.04
charlesmyeri	0	78	4.36	0.24	-1.43	0.26	-1.35	0.14	-1.97
chloris	0	62	4.13	0.26	-1.35	0.25	-1.37	0.18	-1.71
chlorocyanus	1	80	4.38	0.28	-1.29	0.26	-1.34	0.17	-1.77
chocorum	0	79	4.37	0.29	-1.24	0.25	-1.40	0.18	-1.71
christophei	1	50	3.91	0.29	-1.25	0.27	-1.31	0.23	-1.47
chrysolepis	0	74	4.30	0.35	-1.04	0.23	-1.48	0.20	-1.61
chrysops	1	119	4.78	0.27	-1.31	0.28	-1.27	0.17	-1.77
clivicola	1	49	3.89	0.29	-1.24	0.28	-1.27	0.18	-1.71
cobanensis	0	50	3.91	0.28	-1.29	0.32	-1.13	0.23	-1.47
coelestinus	1	84	4.43	0.25	-1.40	0.27	-1.30	0.19	-1.66
compressicauda	0	55	4.01	0.31	-1.17	0.27	-1.31	0.18	-1.71
concolor	1	80	4.38	0.28	-1.26	0.27	-1.31	0.21	-1.56
confusus	1	49	3.89	0.29	-1.24	0.27	-1.31	0.20	-1.61
conspersus	1	70	4.25	0.25	-1.37	0.27	-1.31	0.17	-1.77
cooki	1	73	4.29	0.30	-1.20	0.28	-1.27	0.18	-1.71
crassulus	0	53	3.97	0.28	-1.28	0.26	-1.35	0.19	-1.69
cristatellus	1	80	4.38	0.31	-1.19	0.28	-1.28	0.19	-1.66
cristifer	0	88	4.48	0.24	-1.42	0.25	-1.40	0.15	-1.90

cryptolimifrons	0	45	3.81	0.31	-1.18	0.26	-1.33	0.19	-1.66
cupeyalensis	1	34	3.53	0.23	-1.45	0.27	-1.31	0.19	-1.66
cupreus	0	55	4.01	0.28	-1.26	0.26	-1.36	0.20	-1.61
cuprinus	0	69	4.23	0.30	-1.22	0.25	-1.40	0.22	-1.51
cuscoensis	0	58	4.06	0.26	-1.36	0.25	-1.41	0.19	-1.66
cusuco	0	46	3.83	0.26	-1.33	0.25	-1.37	0.17	-1.77
cuvieri	1	137	4.92	0.28	-1.26	0.29	-1.22	0.19	-1.66
cybotes	1	81	4.39	0.31	-1.18	0.30	-1.21	0.21	-1.56
danieli	0	117	4.76	0.28	-1.26	0.29	-1.25	0.22	-1.51
datzorum	0	47	3.85	0.26	-1.35	0.26	-1.35	0.19	-1.69
desechensis	1	57	4.04	0.31	-1.16	0.28	-1.26	0.21	-1.56
desiradei	1	80	4.38	0.29	-1.24	0.29	-1.24	0.18	-1.71
dissimilis	0	56	4.03	0.22	-1.54	0.28	-1.27	0.14	-1.97
distichus	1	58	4.06	0.29	-1.23	0.25	-1.39	0.19	-1.69
dolichocephalus	1	52	3.95	0.28	-1.29	0.33	-1.10	0.20	-1.61
dollfusianus	0	43	3.76	0.28	-1.28	0.26	-1.36	0.19	-1.66
dominicensis	1	58	4.06	0.28	-1.26	0.24	-1.43	0.18	-1.73
duellmani	0	38	3.64	0.29	-1.23	0.26	-1.34	0.18	-1.71
dunni	0	58	4.06	0.26	-1.33	0.24	-1.42	0.19	-1.66
equestris	1	188	5.24	0.26	-1.35	0.29	-1.23	0.16	-1.83
ernestwilliamsi	1	82	4.41	0.30	-1.20	0.28	-1.26	0.18	-1.71
etheridgei	1	44	3.78	0.35	-1.06	0.28	-1.29	0.22	-1.51
eugenegrahami	1	72	4.28	0.33	-1.12	0.23	-1.45	0.22	-1.51
eulaemus	0	101	4.62	0.29	-1.24	0.26	-1.36	0.23	-1.47
evermanni	1	78	4.36	0.28	-1.29	0.27	-1.29	0.18	-1.71
extremus	1	83	4.42	0.25	-1.37	0.26	-1.34	0.20	-1.61
fairchildi	1	76	4.33	0.22	-1.50	0.30	-1.22	0.16	-1.83
fasciatus	0	72	4.28	0.28	-1.29	0.23	-1.49	0.23	-1.47
favillarum	1	52	3.95	0.30	-1.20	0.25	-1.39	0.19	-1.69

ferreus	1	119	4.78	0.28	-1.28	0.29	-1.23	0.20	-1.61
festae	0	55	4.01	0.22	-1.51	0.28	-1.27	0.16	-1.83
fitchi	0	91	4.51	0.30	-1.21	0.25	-1.41	0.26	-1.35
forresti	1	52	3.95	0.27	-1.31	0.28	-1.27	0.18	-1.71
fortunensis	0	49	3.89	0.26	-1.36	0.25	-1.40	0.17	-1.78
fowleri	1	78	4.36	0.28	-1.27	0.25	-1.38	0.20	-1.61
fraseri	0	109	4.69	0.23	-1.45	0.25	-1.37	0.16	-1.83
frenatus	0	143	4.96	0.30	-1.21	0.25	-1.41	0.21	-1.56
fugitivus	1	35	3.56	0.29	-1.24	0.29	-1.23	0.18	-1.71
fungosus	0	48	3.87	0.24	-1.44	0.25	-1.39	0.13	-2.04
fuscoauratus	0	46	3.83	0.28	-1.28	0.25	-1.38	0.17	-1.77
gadovii	0	80	4.38	0.30	-1.19	0.25	-1.40	0.19	-1.66
gaigei	0	53	3.97	0.29	-1.24	0.23	-1.47	0.18	-1.71
garmani	1	131	4.88	0.26	-1.34	0.28	-1.27	0.17	-1.77
garridoi	1	42	3.74	0.20	-1.61	0.27	-1.33	0.12	-2.12
gemmosus	0	66	4.19	0.29	-1.25	0.25	-1.40	0.25	-1.39
ginaelisae	0	112	4.72	0.25	-1.39	0.25	-1.39	0.18	-1.71
gingivinus	1	72	4.28	0.29	-1.25	0.31	-1.17	0.19	-1.66
gorgonae	1	62	4.13	0.28	-1.29	0.24	-1.43	0.20	-1.61
gracilipes	0	58	4.06	0.33	-1.12	0.24	-1.41	0.21	-1.56
grahami	1	75	4.32	0.27	-1.31	0.29	-1.25	0.17	-1.77
granuliceps	0	47	3.85	0.31	-1.17	0.24	-1.42	0.21	-1.56
griseus	1	136	4.91	0.28	-1.28	0.26	-1.33	0.23	-1.47
gruuo	0	44	3.78	0.27	-1.32	0.25	-1.40	0.17	-1.77
guamuhaya	1	162	5.09	0.21	-1.57	0.29	-1.24	0.13	-2.04
guazuma	1	47	3.85	0.18	-1.70	0.32	-1.15	0.13	-2.04
gundlachi	1	72	4.28	0.31	-1.17	0.29	-1.23	0.21	-1.56
haetianus	1	81	4.39	0.30	-1.20	0.31	-1.18	0.22	-1.51
hendersoni	1	52	3.95	0.26	-1.33	0.31	-1.16	0.19	-1.66

heterodermus	0	76	4.33	0.20	-1.59	0.27	-1.30	0.13	-2.04
heteropholidotus	0	51	3.93	0.29	-1.24	0.25	-1.39	0.19	-1.66
hobartsmithi	0	48	3.87	0.31	-1.17	0.26	-1.35	0.23	-1.49
homolechis	1	70	4.25	0.27	-1.30	0.27	-1.31	0.18	-1.71
huilae	0	80	4.38	0.25	-1.39	0.25	-1.38	0.19	-1.66
humilis	0	44	3.78	0.31	-1.17	0.27	-1.33	0.20	-1.61
ibanezi	0	81	4.39	0.32	-1.14	0.23	-1.47	0.19	-1.66
ignigularis	1	55	4.01	0.31	-1.19	0.27	-1.31	0.19	-1.66
imias	1	65	4.17	0.31	-1.17	0.25	-1.39	0.18	-1.71
inexpectatus	1	37	3.61	0.29	-1.24	0.29	-1.23	0.18	-1.71
insignis	0	157	5.06	0.25	-1.39	0.25	-1.39	0.17	-1.77
insolitus	1	47	3.85	0.21	-1.54	0.28	-1.26	0.13	-2.04
isolepis	1	52	3.95	0.22	-1.53	0.28	-1.26	0.12	-2.12
jacare	0	73	4.29	0.24	-1.43	0.26	-1.34	0.21	-1.56
johnmeyeri	0	73	4.29	0.33	-1.11	0.27	-1.31	0.21	-1.56
juangundlachi	1	36	3.58	0.25	-1.39	0.27	-1.31	0.18	-1.71
jubar	1	62	4.13	0.29	-1.24	0.27	-1.30	0.18	-1.71
kahouannensis	1	73	4.29	0.28	-1.26	0.29	-1.24	0.18	-1.71
kemptoni	0	53	3.97	0.26	-1.35	0.25	-1.37	0.17	-1.77
koopmani	1	39	3.66	0.28	-1.27	0.27	-1.29	0.20	-1.61
krugi	1	55	4.01	0.29	-1.24	0.27	-1.29	0.19	-1.66
kunayalae	0	109	4.69	0.28	-1.29	0.25	-1.37	0.16	-1.83
laeviventris	0	46	3.83	0.24	-1.42	0.26	-1.34	0.16	-1.83
latifrons	0	133	4.89	0.31	-1.16	0.25	-1.37	0.23	-1.47
leachii	1	113	4.73	0.28	-1.26	0.30	-1.21	0.18	-1.71
lemurinus	0	79	4.37	0.30	-1.20	0.26	-1.35	0.21	-1.58
limifrons	0	48	3.87	0.33	-1.12	0.25	-1.38	0.20	-1.63
lineatopus	1	73	4.29	0.29	-1.23	0.30	-1.21	0.21	-1.56
lineatus	1	73	4.29	0.28	-1.26	0.26	-1.35	0.20	-1.61

liogaster	0	54	3.99	0.28	-1.27	0.25	-1.37	0.20	-1.61
lionotus	0	77	4.34	0.31	-1.16	0.24	-1.45	0.18	-1.71
litoralis	1	40	3.69	0.23	-1.47	0.26	-1.36	0.15	-1.90
lividus	1	82	4.41	0.27	-1.30	0.28	-1.27	0.20	-1.61
longiceps	1	83	4.42	0.25	-1.40	0.33	-1.09	0.20	-1.61
longitibialis	1	72	4.28	0.32	-1.13	0.29	-1.24	0.21	-1.56
loveridgei	0	118	4.77	0.30	-1.20	0.25	-1.38	0.22	-1.51
loysianus	1	41	3.71	0.23	-1.45	0.27	-1.32	0.16	-1.83
luciae	1	91	4.51	0.26	-1.35	0.25	-1.39	0.20	-1.61
lucius	1	69	4.23	0.31	-1.16	0.27	-1.31	0.19	-1.66
luteogularis	1	191	5.25	0.25	-1.40	0.30	-1.21	0.17	-1.77
luteosignifer	1	55	4.01	0.27	-1.31	0.25	-1.40	0.19	-1.66
lynchi	0	62	4.13	0.34	-1.08	0.23	-1.49	0.22	-1.51
lyra	0	77	4.34	0.31	-1.18	0.23	-1.46	0.22	-1.51
macilentus	1	41	3.71	0.30	-1.20	0.27	-1.30	0.18	-1.71
macrinii	0	95	4.55	0.26	-1.35	0.25	-1.39	0.19	-1.66
macrolepis	0	62	4.13	0.31	-1.18	0.23	-1.47	0.18	-1.71
macrophallus	0	48	3.87	0.31	-1.17	0.26	-1.35	0.20	-1.61
maculigula	0	107	4.67	0.31	-1.17	0.25	-1.39	0.21	-1.56
maculiventris	0	46	3.83	0.29	-1.23	0.24	-1.42	0.21	-1.56
magnaphallus	0	55	4.01	0.31	-1.18	0.25	-1.38	0.20	-1.61
marcanoi	1	60	4.09	0.31	-1.18	0.30	-1.20	0.18	-1.71
mariarum	0	52	3.95	0.27	-1.32	0.26	-1.35	0.18	-1.71
marmoratus	1	84	4.43	0.27	-1.30	0.27	-1.32	0.20	-1.61
marron	1	50	3.91	0.27	-1.33	0.25	-1.38	0.19	-1.66
marsupialis	0	45	3.81	0.31	-1.16	0.28	-1.26	0.19	-1.66
matudai	0	51	3.93	0.33	-1.11	0.27	-1.31	0.22	-1.51
maynardi	1	76	4.33	0.24	-1.41	0.32	-1.13	0.16	-1.83
medemi	0	52	3.95	0.29	-1.23	0.24	-1.44	0.16	-1.83

megalopithecus	0	88	4.48	0.30	-1.20	0.24	-1.43	0.22	-1.53
megapholidotus	0	53	3.97	0.25	-1.38	0.25	-1.39	0.14	-1.97
menta	0	56	4.03	0.23	-1.48	0.28	-1.28	0.16	-1.83
meridionalis	0	58	4.06	0.25	-1.38	0.23	-1.47	0.19	-1.66
microlepidotus	0	44	3.78	0.25	-1.40	0.23	-1.47	0.16	-1.83
microtus	0	120	4.79	0.23	-1.45	0.26	-1.34	0.18	-1.71
milleri	0	37	3.61	0.31	-1.16	0.29	-1.24	0.21	-1.56
monensis	1	57	4.04	0.29	-1.23	0.29	-1.24	0.20	-1.61
monteverde	0	50	3.91	0.25	-1.38	0.25	-1.40	0.17	-1.77
monticola	1	55	4.01	0.34	-1.09	0.27	-1.31	0.24	-1.43
morazani	0	52	3.95	0.32	-1.16	0.28	-1.27	0.21	-1.56
naufragus	0	50	3.91	0.33	-1.12	0.25	-1.40	0.22	-1.51
neblininus	0	64	4.16	0.23	-1.47	0.29	-1.24	0.15	-1.90
nebuloides	0	51	3.93	0.27	-1.30	0.28	-1.27	0.17	-1.77
nebulosus	0	47	3.85	0.25	-1.37	0.26	-1.35	0.16	-1.83
nelsoni	1	70	4.25	0.29	-1.23	0.26	-1.35	0.23	-1.47
nicefori	0	63	4.14	0.18	-1.74	0.29	-1.25	0.13	-2.04
noblei	1	190	5.25	0.27	-1.33	0.31	-1.18	0.16	-1.83
notopholis	0	49	3.89	0.32	-1.13	0.26	-1.37	0.21	-1.56
nubilus	1	81	4.39	0.29	-1.24	0.28	-1.27	0.21	-1.56
occultus	1	42	3.74	0.17	-1.75	0.26	-1.36	0.10	-2.30
ocelloscapularis	0	42	3.74	0.35	-1.04	0.27	-1.30	0.21	-1.56
oculatus	1	96	4.56	0.28	-1.26	0.28	-1.26	0.21	-1.56
oligaspis	1	54	3.99	0.20	-1.61	0.29	-1.24	0.13	-2.04
olssoni	1	50	3.91	0.26	-1.34	0.25	-1.38	0.21	-1.56
omiltemanus	0	44	3.78	0.23	-1.46	0.26	-1.35	0.17	-1.77
onca	0	92	4.52	0.26	-1.35	0.26	-1.35	0.18	-1.71
opalinus	1	56	4.03	0.26	-1.34	0.27	-1.33	0.18	-1.71
ophiolepis	1	40	3.69	0.22	-1.51	0.25	-1.39	0.21	-1.56

orcesi	0	59	4.08	0.19	-1.66	0.27	-1.33	0.13	-2.04
ortonii	0	59	4.08	0.26	-1.36	0.25	-1.38	0.18	-1.71
oxylophus	0	76	4.33	0.29	-1.24	0.24	-1.43	0.17	-1.77
pachypus	0	54	3.99	0.31	-1.17	0.24	-1.41	0.20	-1.61
parilis	0	81	4.39	0.27	-1.31	0.26	-1.35	0.15	-1.90
parvauritus	0	86	4.45	0.27	-1.31	0.27	-1.31	0.21	-1.56
parvicirculatus	0	50	3.91	0.35	-1.06	0.27	-1.30	0.20	-1.61
paternus	1	50	3.91	0.22	-1.51	0.29	-1.23	0.15	-1.90
pentaprion	0	77	4.34	0.24	-1.44	0.26	-1.36	0.13	-2.04
peraccae	0	52	3.95	0.25	-1.38	0.25	-1.39	0.22	-1.51
petersii	0	118	4.77	0.26	-1.35	0.24	-1.42	0.18	-1.71
peucephilus	0	41	3.71	0.22	-1.51	0.25	-1.39	0.15	-1.90
philopunctatus	0	73	4.29	0.27	-1.32	0.27	-1.30	0.18	-1.71
pigmaequestris	1	140	4.94	0.26	-1.34	0.29	-1.23	0.17	-1.77
pinchoti	1	52	3.95	0.28	-1.27	0.28	-1.29	0.21	-1.56
placidus	1	46	3.83	0.17	-1.75	0.28	-1.29	0.10	-2.30
planiceps	0	76	4.33	0.32	-1.14	0.23	-1.45	0.20	-1.61
poecilopus	0	72	4.28	0.32	-1.14	0.23	-1.49	0.19	-1.66
pogus	1	50	3.91	0.27	-1.30	0.29	-1.22	0.21	-1.56
polylepis	0	57	4.04	0.31	-1.18	0.26	-1.35	0.22	-1.51
poncensis	1	49	3.89	0.24	-1.42	0.27	-1.29	0.18	-1.71
porcatus	1	78	4.36	0.24	-1.41	0.37	-1.00	0.17	-1.77
porcus	1	162	5.09	0.22	-1.51	0.29	-1.23	0.14	-1.97
princeps	0	117	4.76	0.32	-1.13	0.24	-1.43	0.22	-1.51
proboscis	0	75	4.32	0.18	-1.70	0.30	-1.21	0.13	-2.04
properus	1	51	3.93	0.28	-1.27	0.26	-1.35	0.19	-1.66
pseudokemptoni	0	55	4.01	0.26	-1.35	0.26	-1.35	0.17	-1.77
pulchellus	1	51	3.93	0.26	-1.35	0.30	-1.21	0.18	-1.74
pumilus	1	33	3.50	0.23	-1.49	0.26	-1.34	0.16	-1.83

punctatus	0	89	4.49	0.25	-1.40	0.27	-1.31	0.17	-1.77
purpurgularis	0	59	4.08	0.33	-1.12	0.27	-1.31	0.22	-1.51
pygmaeus	0	35	3.56	0.30	-1.20	0.27	-1.31	0.19	-1.66
quadriocellifer	1	56	4.03	0.26	-1.35	0.25	-1.40	0.19	-1.66
quaggulus	0	40	3.69	0.32	-1.15	0.29	-1.25	0.20	-1.63
quercorum	0	45	3.81	0.25	-1.39	0.25	-1.39	0.16	-1.83
ravitergum	1	56	4.03	0.28	-1.28	0.25	-1.39	0.19	-1.66
reconditus	1	100	4.61	0.31	-1.18	0.30	-1.22	0.22	-1.51
rejectus	1	37	3.61	0.28	-1.29	0.26	-1.35	0.16	-1.83
richardi	1	140	4.94	0.29	-1.25	0.24	-1.41	0.21	-1.56
ricordii	1	160	5.08	0.26	-1.35	0.28	-1.26	0.18	-1.71
rimarum	1	45	3.81	0.34	-1.07	0.27	-1.31	0.21	-1.56
rivalis	0	64	4.16	0.34	-1.07	0.23	-1.49	0.20	-1.61
roatanensis	1	51	3.93	0.34	-1.08	0.26	-1.35	0.21	-1.56
rodriguezii	0	46	3.83	0.32	-1.13	0.25	-1.39	0.17	-1.77
roosevelti	1	160	5.08	0.31	-1.17	0.30	-1.20	0.18	-1.71
roquet	1	86	4.45	0.27	-1.33	0.25	-1.38	0.18	-1.71
rubiginosus	0	49	3.89	0.28	-1.27	0.26	-1.35	0.21	-1.56
rubribarbaris	0	48	3.87	0.30	-1.20	0.28	-1.27	0.20	-1.63
rubribarbus	1	63	4.14	0.30	-1.20	0.27	-1.32	0.20	-1.61
ruizii	0	57	4.04	0.23	-1.47	0.27	-1.29	0.17	-1.80
rupinae	1	56	4.03	0.32	-1.15	0.28	-1.27	0.23	-1.47
sabanus	1	69	4.23	0.28	-1.26	0.28	-1.27	0.18	-1.71
sagrei	1	70	4.25	0.27	-1.29	0.26	-1.37	0.21	-1.56
salvini	0	61	4.11	0.22	-1.49	0.26	-1.33	0.16	-1.83
schiedii	0	58	4.06	0.33	-1.11	0.27	-1.31	0.25	-1.40
schwartzi	1	49	3.89	0.30	-1.21	0.28	-1.29	0.22	-1.51
scriptus	1	76	4.33	0.29	-1.25	0.26	-1.34	0.21	-1.56
scypheus	0	78	4.36	0.33	-1.10	0.23	-1.45	0.21	-1.56

semilineatus	1	47	3.85	0.28	-1.27	0.28	-1.28	0.19	-1.66
sericeus	0	47	3.85	0.26	-1.35	0.23	-1.45	0.15	-1.90
serranoi	0	85	4.44	0.30	-1.20	0.25	-1.40	0.21	-1.56
sheplani	1	41	3.71	0.16	-1.83	0.27	-1.33	0.10	-2.33
shrevei	1	60	4.09	0.29	-1.24	0.28	-1.27	0.20	-1.61
smallwoodi	1	190	5.25	0.26	-1.34	0.29	-1.25	0.16	-1.83
smaragdinus	1	57	4.04	0.23	-1.46	0.29	-1.22	0.14	-1.97
sminthus	0	52	3.95	0.27	-1.29	0.24	-1.42	0.21	-1.56
soinii	0	82	4.41	0.29	-1.23	0.24	-1.42	0.22	-1.51
solitarius	0	52	3.95	0.22	-1.50	0.26	-1.35	0.17	-1.77
spectrum	1	42	3.74	0.26	-1.35	0.27	-1.31	0.15	-1.90
squamulatus	0	95	4.55	0.26	-1.33	0.27	-1.30	0.24	-1.43
strahmi	1	80	4.38	0.33	-1.10	0.28	-1.29	0.18	-1.71
stratulus	1	52	3.95	0.26	-1.35	0.29	-1.25	0.17	-1.80
subocularis	0	63	4.14	0.28	-1.26	0.25	-1.39	0.18	-1.71
sulcifrons	0	62	4.13	0.24	-1.42	0.24	-1.44	0.18	-1.71
tandai	0	70	4.25	0.38	-0.97	0.23	-1.47	0.20	-1.61
taylori	0	78	4.36	0.28	-1.28	0.24	-1.42	0.19	-1.66
terraealtae	1	80	4.38	0.29	-1.24	0.28	-1.26	0.19	-1.68
terueli	1	39	3.66	0.22	-1.52	0.26	-1.35	0.16	-1.83
tigrinus	0	57	4.04	0.22	-1.52	0.26	-1.36	0.13	-2.04
tolimensis	0	55	4.01	0.27	-1.31	0.26	-1.36	0.20	-1.61
townsendi	1	57	4.04	0.28	-1.28	0.26	-1.36	0.23	-1.47
trachyderma	0	61	4.11	0.32	-1.12	0.21	-1.54	0.20	-1.61
transversalis	0	84	4.43	0.25	-1.37	0.24	-1.41	0.18	-1.71
trinitatis	1	74	4.30	0.25	-1.37	0.25	-1.40	0.16	-1.83
tropidogaster	0	55	4.01	0.30	-1.20	0.25	-1.39	0.20	-1.61
tropidolepis	0	59	4.08	0.30	-1.21	0.24	-1.44	0.21	-1.56
tropidonotus	0	55	4.01	0.30	-1.21	0.24	-1.41	0.20	-1.61

uniformis	0	40	3.69	0.32	-1.13	0.28	-1.29	0.18	-1.71	
unilobatus	0	49	3.89	0.24	-1.41	0.25	-1.38	0.17	-1.77	
utilensis	1	62	4.13	0.26	-1.35	0.25	-1.39	0.15	-1.90	
valencienni	1	86	4.45	0.22	-1.53	0.29	-1.24	0.13	-2.04	
vanidicus	1	39	3.66	0.24	-1.43	0.29	-1.24	0.16	-1.83	
vanzolinii	0	104	4.64	0.20	-1.61	0.26	-1.33	0.14	-1.97	
vaupesianus	0	82	4.41	0.23	-1.48	0.26	-1.35	0.18	-1.71	
ventrimaculatus	0	80	4.38	0.34	-1.08	0.27	-1.32	0.26	-1.35	
vermiculatus	1	123	4.81	0.30	-1.22	0.28	-1.26	0.18	-1.71	
villai	1	55	4.01	0.29	-1.23	0.25	-1.38	0.21	-1.56	
vittigerus	0	60	4.09	0.31	-1.17	0.25	-1.39	0.20	-1.61	
wattsi	1	58	4.06	0.28	-1.29	0.28	-1.26	0.21	-1.56	
websteri	1	55	4.01	0.26	-1.35	0.24	-1.44	0.17	-1.77	
wellbornae	0	50	3.91	0.25	-1.39	0.24	-1.43	0.16	-1.83	
whitemani	1	67	4.20	0.31	-1.18	0.30	-1.22	0.19	-1.66	
williamsmittermeierorun	0	66	4.19	0.21	-1.57	0.27	-1.30	0.14	-1.97	
woodi	0	92	4.52	0.31	-1.18	0.26	-1.35	0.20	-1.61	
yoroensis	0	39	3.66	0.31	-1.17	0.27	-1.31	0.19	-1.66	
zeus	0	43	3.76	0.34	-1.08	0.24	-1.43	0.20	-1.61	

	TAL/SVL	lntal	VSC	lnvsc	DSC	lndsc	LAM	lnlam	HSC	lnhsc
acutus	1.83	0.60	7.0	1.95	11.0	2.40	23.0	3.14	7.0	1.95
aeneus	1.23	0.21	7.0	1.95	11.0	2.40	24.0	3.18	8.0	2.08
aequatorialis	2.66	0.98	10.0	2.30	11.0	2.40	23.0	3.14	13.0	2.56
agassizi	1.70	0.53	9.0	2.20	10.0	2.30	34.0	3.53	7.0	1.95
agueroi	0.92	-0.08	9.0	2.20	3.0	1.10	29.0	3.37	7.0	1.95
ahli	1.61	0.48	7.0	1.95	11.0	2.40	16.0	2.77	9.0	2.20
aliniger	1.45	0.37	6.0	1.79	8.0	2.08	23.0	3.14	8.0	2.08
allisoni	2.08	0.73	8.0	2.08	8.0	2.08	29.0	3.37	7.0	1.95

allogus	1.36	0.31	8.0	2.08	10.0	2.30	16.0	2.77	10.0	2.30
altae	2.20	0.79	7.8	2.05	12.0	2.48	15.0	2.71	8.6	2.15
altavelensis	1.50	0.41	6.0	1.79	12.0	2.48	19.0	2.94	6.0	1.79
altitudinalis	1.38	0.32	6.0	1.79	8.0	2.08	24.0	3.18	5.0	1.61
alumina	2.80	1.03	4.0	1.39	6.0	1.79	16.0	2.77	7.0	1.95
alutaceus	2.20	0.79	7.0	1.95	7.0	1.95	17.0	2.83	8.0	2.08
alvarezdeltoroi	1.96	0.67	9.0	2.20	11.0	2.40	18.0	2.89	8.0	2.08
amplisquamosus	2.35	0.85	4.0	1.39	3.5	1.25	16.0	2.77	5.0	1.61
anchicayae	2.10	0.74	8.0	2.08	7.0	1.95	17.0	2.83	11.0	2.40
anfiloquiae	2.71	1.00	8.0	2.08	8.0	2.08	17.0	2.83	8.0	2.08
angusticeps	0.91	-0.09	9.0	2.20	15.0	2.71	19.0	2.94	7.0	1.95
annectens	2.24	0.81	3.0	1.10	5.0	1.61	14.0	2.64	10.0	2.30
antioquiae	2.30	0.83	10.0	2.30	13.0	2.56	21.5	3.07	18.0	2.89
antonii	2.10	0.74	7.0	1.95	10.0	2.30	17.0	2.83	9.0	2.20
apletophallus	2.10	0.74	6.5	1.87	12.0	2.48	17.0	2.83	12.5	2.53
apollinaris	2.74	1.01	9.0	2.20	10.0	2.30	26.0	3.26	11.0	2.40
aquaticus	1.63	0.49	7.0	1.95	7.0	1.95	16.0	2.77	12.0	2.48
argenteolus	1.89	0.64	10.0	2.30	12.0	2.48	23.0	3.14	8.0	2.08
argillaceus	1.84	0.61	5.0	1.61	9.0	2.20	18.0	2.89	5.0	1.61
armouri	1.71	0.54	4.0	1.39	6.0	1.79	17.0	2.83	7.0	1.95
auratus	2.65	0.97	3.0	1.10	3.0	1.10	15.0	2.71	9.0	2.20
bahorucoensis	2.73	1.00	6.5	1.87	8.0	2.08	19.0	2.94	8.0	2.08
baleatus	1.98	0.68	9.0	2.20	6.0	1.79	32.0	3.47	5.0	1.61
baracoae	1.75	0.56	8.0	2.08	6.0	1.79	47.0	3.85	6.0	1.79
barahonae	1.83	0.60	8.0	2.08	6.0	1.79	35.0	3.56	6.0	1.79
barbatus	0.95	-0.05	12.0	2.48	3.0	1.10	34.0	3.53	7.0	1.95
barbouri	2.33	0.85	3.0	1.10	3.0	1.10	10.0	2.30	7.0	1.95
barkeri	2.00	0.69	8.7	2.16	8.7	2.16	16.0	2.77	9.0	2.20
bartschi	1.92	0.65	10.0	2.30	12.0	2.48	26.0	3.26	9.0	2.20

beckeri	1.50	0.41	7.0	1.95	10.0	2.30	20.0	3.00	8.0	2.08
bellipeniculus	1.39	0.33	6.0	1.79	7.0	1.95	22.0	3.09	5.0	1.61
benedikti	1.70	0.53	6.0	1.79	8.0	2.08	14.0	2.64	15.0	2.71
bicaorum	1.82	0.60	6.0	1.79	8.0	2.08	17.0	2.83	10.0	2.30
bimaculatus	1.85	0.62	7.0	1.95	6.0	1.79	29.0	3.37	7.0	1.95
binotatus	2.00	0.69	5.0	1.61	5.0	1.61	15.0	2.71	11.0	2.40
biporcatus	2.10	0.74	5.2	1.65	8.0	2.08	22.1	3.10	10.0	2.30
biscutiger	1.89	0.64	8.0	2.08	11.0	2.40	16.0	2.77	11.0	2.40
blanquillanus	1.89	0.64	5.0	1.61	8.0	2.08	27.0	3.30	7.0	1.95
bocourti	1.95	0.67	8.5	2.14	11.0	2.40	17.0	2.83	11.3	2.42
boettgeri	2.50	0.92	7.0	1.95	10.0	2.30	19.0	2.94	9.0	2.20
bombiceps	1.87	0.63	4.5	1.50	7.5	2.01	15.0	2.71	12.0	2.48
bonairensis	2.15	0.77	6.0	1.79	11.0	2.40	26.0	3.26	8.0	2.08
boulengerianus	2.00	0.69	5.0	1.61	5.0	1.61	14.0	2.64	8.0	2.08
brasiliensis	2.53	0.93	4.5	1.50	6.0	1.79	17.5	2.86	9.5	2.25
bremeri	1.53	0.43	5.0	1.61	7.0	1.95	19.0	2.94	6.0	1.79
breslini	1.65	0.50	5.0	1.61	7.0	1.95	16.0	2.77	6.0	1.79
brevirostris	1.45	0.37	7.0	1.95	12.0	2.48	17.0	2.83	6.0	1.79
brunneus	2.00	0.69	6.0	1.79	6.0	1.79	21.0	3.04	5.0	1.61
calimae	1.52	0.42	10.0	2.30	12.0	2.48	17.0	2.83	9.0	2.20
campbelli	1.26	0.23	5.0	1.61	7.0	1.95	16.0	2.77	7.3	1.99
capito	1.87	0.63	6.0	1.79	6.0	1.79	17.0	2.83	11.0	2.40
caquetae	1.90	0.64	8.0	2.08	12.0	2.48	22.0	3.09	10.0	2.30
carlostoddi	1.39	0.33	5.0	1.61	7.0	1.95	18.0	2.89	5.0	1.61
carolinensis	1.92	0.65	7.0	1.95	6.0	1.79	24.0	3.18	7.0	1.95
carpenteri	1.61	0.48	7.0	1.95	9.0	2.20	16.0	2.77	13.0	2.56
casildae	2.80	1.03	11.0	2.40	9.0	2.20	23.0	3.14	16.0	2.77
caudalis	1.34	0.29	8.0	2.08	13.0	2.56	16.0	2.77	6.0	1.79
centralis	1.50	0.41	5.0	1.61	8.0	2.08	17.0	2.83	5.0	1.61

chamaeleonides	1.10	0.10	13.0	2.56	3.0	1.10	35.0	3.56	9.0	2.20
charlesmyeri	1.03	0.03	9.0	2.20	10.0	2.30	23.0	3.14	7.5	2.01
chloris	2.01	0.70	9.0	2.20	13.0	2.56	18.0	2.89	13.0	2.56
chlorocyanus	2.03	0.71	6.5	1.87	7.0	1.95	29.0	3.37	7.0	1.95
chocorum	2.38	0.87	10.0	2.30	12.0	2.48	20.0	3.00	13.0	2.56
christophei	1.78	0.58	8.0	2.08	9.0	2.20	20.0	3.00	9.0	2.20
chrysolepis	2.00	0.69	4.5	1.50	5.0	1.61	16.0	2.77	11.5	2.44
chrysops	1.67	0.51	6.0	1.79	8.0	2.08	25.0	3.22	6.0	1.79
clivicola	2.20	0.79	9.0	2.20	6.0	1.79	17.0	2.83	9.0	2.20
cobanensis	1.78	0.58	7.0	1.95	8.0	2.08	17.0	2.83	10.0	2.30
coelestinus	1.85	0.62	6.5	1.87	8.0	2.08	27.0	3.30	11.0	2.40
compressicauda	1.65	0.50	4.0	1.39	4.0	1.39	15.0	2.71	8.0	2.08
concolor	1.91	0.65	6.0	1.79	7.0	1.95	21.0	3.04	9.0	2.20
confusus	1.50	0.41	6.0	1.79	11.0	2.40	18.0	2.89	5.0	1.61
conspersus	2.00	0.69	9.0	2.20	13.0	2.56	23.0	3.14	8.0	2.08
cooki	2.10	0.74	7.0	1.95	9.0	2.20	18.0	2.89	6.0	1.79
crassulus	1.95	0.67	3.0	1.10	4.0	1.39	16.0	2.77	7.0	1.95
cristatellus	1.62	0.48	7.0	1.95	7.0	1.95	20.0	3.00	6.0	1.79
cristifer	1.27	0.24	8.0	2.08	10.0	2.30	23.0	3.14	9.0	2.20
cryptolimifrons	1.61	0.48	6.5	1.87	11.0	2.40	16.5	2.80	12.5	2.53
cupeyalensis	3.00	1.10	5.0	1.61	4.0	1.39	16.0	2.77	6.0	1.79
cupreus	1.92	0.65	6.0	1.79	6.0	1.79	15.0	2.71	10.0	2.30
cuprinus	1.20	0.18	7.4	2.00	6.0	1.79	16.0	2.77	9.0	2.20
cuscoensis	2.66	0.98	7.0	1.95	10.0	2.30	17.0	2.83	10.0	2.30
cusuco	1.63	0.49	5.0	1.61	7.0	1.95	16.0	2.77	8.5	2.14
cuvieri	2.25	0.81	8.0	2.08	5.0	1.61	32.0	3.47	9.0	2.20
cybotes	1.69	0.52	5.0	1.61	7.0	1.95	19.0	2.94	7.0	1.95
danieli	2.39	0.87	10.0	2.30	7.0	1.95	24.0	3.18	10.0	2.30
datzorum	1.40	0.34	7.0	1.95	10.0	2.30	16.5	2.80	7.5	2.01

desechensis	2.14	0.76	6.0	1.79	7.0	1.95	23.0	3.14	6.0	1.79
desiradei	1.70	0.53	7.0	1.95	7.0	1.95	25.0	3.22	5.5	1.70
dissimilis	1.88	0.63	8.0	2.08	9.5	2.25	16.0	2.77	7.0	1.95
distichus	1.30	0.26	6.0	1.79	13.0	2.56	18.0	2.89	6.0	1.79
dolichocephalus	2.60	0.96	6.0	1.79	9.0	2.20	22.0	3.09	8.0	2.08
dollfusianus	2.10	0.74	5.5	1.70	6.5	1.87	14.0	2.64	11.0	2.40
dominicensis	1.15	0.14	7.0	1.95	14.5	2.67	21.0	3.04	4.0	1.39
duellmani	1.63	0.49	4.5	1.50	4.0	1.39	13.0	2.56	7.0	1.95
dunni	1.94	0.66	6.0	1.79	7.0	1.95	17.0	2.83	6.0	1.79
equestris	1.95	0.67	7.0	1.95	5.0	1.61	32.0	3.47	6.0	1.79
ernestwilliamsi	1.70	0.53	8.0	2.08	9.0	2.20	25.0	3.22	7.0	1.95
etheridgei	2.26	0.82	6.0	1.79	8.0	2.08	19.0	2.94	12.0	2.48
eugenegrahami	2.17	0.77	11.0	2.40	10.0	2.30	25.0	3.22	14.0	2.64
eulaemus	2.02	0.70	11.0	2.40	11.0	2.40	22.0	3.09	16.0	2.77
evermanni	1.43	0.36	7.0	1.95	9.0	2.20	25.0	3.22	7.0	1.95
extremus	1.32	0.28	7.0	1.95	9.0	2.20	25.0	3.22	9.0	2.20
fairchildi	2.05	0.72	7.0	1.95	6.0	1.79	27.0	3.30	5.0	1.61
fasciatus	2.40	0.88	9.0	2.20	11.0	2.40	21.0	3.04	11.0	2.40
favillarum	1.50	0.41	7.5	2.01	13.5	2.60	21.0	3.04	4.0	1.39
ferreus	1.95	0.67	6.0	1.79	5.0	1.61	29.0	3.37	5.0	1.61
festae	2.00	0.69	9.0	2.20	9.0	2.20	18.0	2.89	10.0	2.30
fitchi	2.20	0.79	10.0	2.30	10.0	2.30	22.0	3.09	17.0	2.83
forresti	1.70	0.53	5.0	1.61	5.0	1.61	20.0	3.00	4.8	1.56
fortunensis	1.96	0.67	9.0	2.20	12.0	2.48	16.0	2.77	11.0	2.40
fowleri	2.17	0.77	10.0	2.30	10.0	2.30	20.0	3.00	7.0	1.95
fraseri	2.40	0.88	8.0	2.08	11.0	2.40	22.0	3.09	8.0	2.08
frenatus	1.58	0.46	10.0	2.30	10.0	2.30	25.0	3.22	12.0	2.48
fugitivus	2.34	0.85	6.0	1.79	4.0	1.39	16.0	2.77	8.0	2.08
fungosus	1.12	0.11	8.5	2.14	10.0	2.30	14.5	2.67	7.0	1.95

fuscoauratus	1.91	0.65	8.0	2.08	13.0	2.56	16.0	2.77	12.0	2.48
gadovii	2.01	0.70	7.0	1.95	8.0	2.08	20.0	3.00	11.0	2.40
gaigei	1.68	0.52	5.0	1.61	7.0	1.95	16.0	2.77	10.9	2.39
garmani	2.01	0.70	8.0	2.08	6.5	1.87	29.0	3.37	9.0	2.20
garridoi	1.11	0.10	7.0	1.95	11.0	2.40	17.0	2.83	6.0	1.79
gemmosus	2.70	0.99	12.0	2.48	12.0	2.48	20.0	3.00	15.0	2.71
ginaelisae	2.19	0.78	9.0	2.20	8.0	2.08	22.0	3.09	7.0	1.95
gingivinus	1.92	0.65	5.0	1.61	7.0	1.95	21.0	3.04	7.0	1.95
gorgonae	2.00	0.69	7.0	1.95	10.0	2.30	18.0	2.89	11.0	2.40
gracilipes	1.97	0.68	7.0	1.95	6.0	1.79	14.0	2.64	13.0	2.56
grahami	1.70	0.53	7.0	1.95	11.0	2.40	25.0	3.22	11.0	2.40
granuliceps	1.63	0.49	6.0	1.79	12.0	2.48	15.0	2.71	18.0	2.89
griseus	2.00	0.69	7.0	1.95	10.0	2.30	28.0	3.33	9.0	2.20
gruuo	1.70	0.53	8.0	2.08	12.0	2.48	16.0	2.77	9.0	2.20
guamuhaya	0.97	-0.03	8.5	2.14	2.5	0.92	33.0	3.50	6.0	1.79
guazuma	0.95	-0.05	7.0	1.95	12.0	2.48	17.0	2.83	6.0	1.79
gundlachi	1.71	0.54	6.0	1.79	8.0	2.08	18.0	2.89	10.0	2.30
haetianus	1.89	0.64	5.0	1.61	6.0	1.79	20.0	3.00	6.0	1.79
hendersoni	2.82	1.04	6.0	1.79	10.0	2.30	20.0	3.00	7.0	1.95
heterodermus	1.47	0.39	6.0	1.79	4.0	1.39	21.0	3.04	5.0	1.61
heteropholidotus	2.00	0.69	5.0	1.61	4.0	1.39	16.0	2.77	5.0	1.61
hobartsmithi	1.87	0.63	5.5	1.70	8.5	2.14	17.0	2.83	10.0	2.30
homolechis	1.59	0.46	5.0	1.61	8.0	2.08	18.0	2.89	7.0	1.95
huilae	2.35	0.85	9.0	2.20	9.0	2.20	22.0	3.09	9.0	2.20
humilis	1.32	0.28	5.0	1.61	4.0	1.39	13.0	2.56	9.0	2.20
ibanezi	2.60	0.96	9.0	2.20	9.0	2.20	19.0	2.94	11.0	2.40
ignigularis	1.57	0.45	6.0	1.79	11.0	2.40	18.0	2.89	4.0	1.39
imias	1.50	0.41	8.0	2.08	9.0	2.20	17.0	2.83	8.0	2.08
inexpectatus	2.20	0.79	8.0	2.08	6.0	1.79	25.0	3.22	8.0	2.08

insignis	2.06	0.72	9.5	2.25	9.5	2.25	27.0	3.30	10.0	2.30
insolitus	1.14	0.13	10.0	2.30	9.0	2.20	16.0	2.77	4.0	1.39
isolepis	1.50	0.41	5.0	1.61	7.0	1.95	21.0	3.04	4.0	1.39
jacare	1.90	0.64	7.0	1.95	10.0	2.30	22.0	3.09	7.0	1.95
johnmeyeri	1.72	0.54	4.0	1.39	5.5	1.70	17.0	2.83	8.5	2.14
juangundlachi	2.41	0.88	5.0	1.61	4.0	1.39	15.0	2.71	7.0	1.95
jubar	1.50	0.41	6.0	1.79	10.0	2.30	19.0	2.94	6.0	1.79
kahouannensis	1.49	0.40	6.0	1.79	8.0	2.08	25.0	3.22	5.0	1.61
kemptoni	1.98	0.68	8.0	2.08	10.0	2.30	16.0	2.77	9.0	2.20
koopmani	2.10	0.74	4.0	1.39	4.0	1.39	17.0	2.83	10.0	2.30
krugi	2.55	0.94	5.0	1.61	6.0	1.79	21.0	3.04	6.0	1.79
kunayalae	1.90	0.64	9.0	2.20	10.0	2.30	13.0	2.56	15.0	2.71
laeviventris	1.78	0.58	6.0	1.79	6.0	1.79	15.0	2.71	8.0	2.08
latifrons	2.23	0.80	11.0	2.40	10.0	2.30	23.0	3.14	13.0	2.56
leachii	1.60	0.47	7.5	2.01	8.0	2.08	31.0	3.43	6.0	1.79
lemurinus	2.04	0.71	6.0	1.79	10.0	2.30	18.0	2.89	9.0	2.20
limifrons	2.10	0.74	6.5	1.87	10.0	2.30	15.0	2.71	13.0	2.56
lineatopus	1.46	0.38	8.0	2.08	10.0	2.30	22.0	3.09	9.0	2.20
lineatus	2.20	0.79	6.0	1.79	7.0	1.95	20.0	3.00	7.0	1.95
liogaster	1.42	0.35	5.0	1.61	5.0	1.61	16.0	2.77	6.0	1.79
lionotus	1.47	0.39	9.0	2.20	5.0	1.61	15.0	2.71	11.0	2.40
litoralis	1.55	0.44	5.0	1.61	9.0	2.20	18.0	2.89	5.0	1.61
lividus	1.72	0.54	7.0	1.95	8.0	2.08	26.0	3.26	6.0	1.79
longiceps	2.00	0.69	8.0	2.08	6.0	1.79	29.0	3.37	7.0	1.95
longitibialis	1.69	0.52	5.0	1.61	6.0	1.79	16.0	2.77	8.0	2.08
loveridgei	1.89	0.64	7.0	1.95	8.0	2.08	26.0	3.26	12.0	2.48
loysianus	1.20	0.18	7.0	1.95	9.0	2.20	17.0	2.83	5.0	1.61
luciae	1.38	0.32	7.0	1.95	12.0	2.48	26.0	3.26	8.0	2.08
lucius	1.90	0.64	8.0	2.08	15.0	2.71	26.0	3.26	7.0	1.95

luteogularis	1.82	0.60	8.0	2.08	5.0	1.61	44.0	3.78	4.0	1.39
luteosignifer	1.50	0.41	5.0	1.61	9.0	2.20	18.0	2.89	6.0	1.79
lynchi	1.97	0.68	10.0	2.30	11.0	2.40	16.0	2.77	23.0	3.14
lyra	2.08	0.73	5.0	1.61	8.0	2.08	19.0	2.94	10.0	2.30
macilentus	2.83	1.04	7.0	1.95	5.0	1.61	18.0	2.89	8.0	2.08
macrinii	2.00	0.69	7.0	1.95	8.0	2.08	25.0	3.22	8.0	2.08
macrolepis	1.50	0.41	8.0	2.08	5.0	1.61	16.0	2.77	10.0	2.30
macrophallus	2.16	0.77	5.0	1.61	6.0	1.79	15.0	2.71	9.0	2.20
maculigula	2.08	0.73	12.0	2.48	7.0	1.95	19.0	2.94	16.0	2.77
maculiventris	1.93	0.66	8.0	2.08	11.0	2.40	16.0	2.77	13.0	2.56
magnaphallus	1.90	0.64	5.5	1.70	6.0	1.79	14.0	2.64	12.0	2.48
marcanoi	2.13	0.76	4.0	1.39	6.0	1.79	19.0	2.94	8.0	2.08
mariarum	2.30	0.83	7.0	1.95	8.0	2.08	16.0	2.77	10.0	2.30
marmoratus	1.80	0.59	8.0	2.08	7.0	1.95	25.0	3.22	6.0	1.79
marron	1.40	0.34	6.0	1.79	11.0	2.40	17.0	2.83	6.0	1.79
marsupialis	1.45	0.37	4.0	1.39	4.0	1.39	13.0	2.56	9.0	2.20
matudai	2.20	0.79	4.0	1.39	6.0	1.79	16.0	2.77	8.0	2.08
maynardi	2.05	0.72	7.0	1.95	7.0	1.95	27.0	3.30	6.0	1.79
medemi	2.00	0.69	9.0	2.20	14.0	2.64	15.0	2.71	14.0	2.64
megalopithecus	2.50	0.92	13.0	2.56	12.0	2.48	21.0	3.04	14.0	2.64
megapholidotus	1.60	0.47	3.0	1.10	3.0	1.10	14.5	2.67	7.5	2.01
menta	1.80	0.59	7.0	1.95	10.0	2.30	19.0	2.94	6.0	1.79
meridionalis	2.36	0.86	3.5	1.25	6.0	1.79	14.0	2.64	9.0	2.20
microlepidotus	1.50	0.41	6.0	1.79	7.0	1.95	15.0	2.71	6.0	1.79
microtus	2.10	0.74	7.0	1.95	8.0	2.08	22.0	3.09	6.0	1.79
milleri	1.84	0.61	7.0	1.95	7.0	1.95	15.0	2.71	8.0	2.08
monensis	2.00	0.69	9.0	2.20	8.0	2.08	20.0	3.00	7.0	1.95
monteverde	1.70	0.53	6.0	1.79	9.0	2.20	15.0	2.71	7.8	2.05
monticola	2.10	0.74	6.0	1.79	6.0	1.79	18.0	2.89	10.0	2.30

morazani	2.57	0.94	4.0	1.39	4.0	1.39	15.0	2.71	5.5	1.70
naufragus	1.89	0.64	7.0	1.95	9.0	2.20	15.0	2.71	7.0	1.95
neblininus	1.50	0.41	7.0	1.95	8.0	2.08	20.0	3.00	5.0	1.61
nebuloides	1.45	0.37	4.5	1.50	6.0	1.79	15.0	2.71	7.0	1.95
nebulosus	2.01	0.70	4.5	1.50	5.5	1.70	13.0	2.56	7.0	1.95
nelsoni	1.95	0.67	5.0	1.61	10.0	2.30	22.0	3.09	8.0	2.08
nicefori	1.30	0.26	6.5	1.87	7.0	1.95	18.0	2.89	6.0	1.79
noblei	2.30	0.83	8.0	2.08	5.0	1.61	39.0	3.66	8.0	2.08
notopholis	2.20	0.79	4.0	1.39	3.0	1.10	13.0	2.56	9.0	2.20
nubilus	2.00	0.69	6.0	1.79	7.0	1.95	28.0	3.33	7.0	1.95
occultus	1.00	0.00	6.0	1.79	12.0	2.48	17.0	2.83	13.0	2.56
ocelloscapularis	2.29	0.83	7.0	1.95	11.0	2.40	16.0	2.77	11.0	2.40
oculatus	1.60	0.47	7.0	1.95	6.0	1.79	24.0	3.18	7.0	1.95
oligaspis	1.19	0.17	9.0	2.20	15.0	2.71	19.0	2.94	9.0	2.20
olssoni	3.00	1.10	4.0	1.39	3.0	1.10	19.0	2.94	7.0	1.95
omiltemanus	1.98	0.68	5.0	1.61	6.0	1.79	16.0	2.77	6.0	1.79
onca	1.99	0.69	6.0	1.79	6.0	1.79	15.0	2.71	10.0	2.30
opalinus	1.80	0.59	6.0	1.79	9.0	2.20	21.0	3.04	8.0	2.08
ophiolepis	1.80	0.59	4.0	1.39	3.0	1.10	15.0	2.71	9.0	2.20
orcesi	1.27	0.24	8.0	2.08	11.0	2.40	18.0	2.89	4.0	1.39
ortonii	1.75	0.56	7.0	1.95	11.0	2.40	18.0	2.89	9.0	2.20
oxylophus	1.74	0.55	7.5	2.01	6.0	1.79	15.5	2.74	10.5	2.35
pachypus	1.80	0.59	6.5	1.87	7.0	1.95	14.0	2.64	14.0	2.64
parilis	2.00	0.69	10.0	2.30	8.0	2.08	16.0	2.77	17.0	2.83
parvauritus	2.05	0.72	4.7	1.55	7.0	1.95	22.3	3.10	11.7	2.46
parvicirculatus	1.88	0.63	7.0	1.95	6.0	1.79	17.0	2.83	9.0	2.20
paternus	0.83	-0.19	8.0	2.08	11.0	2.40	18.0	2.89	6.0	1.79
pentaprion	1.26	0.23	8.5	2.14	12.0	2.48	21.0	3.04	7.0	1.95
peraccae	2.24	0.81	9.0	2.20	11.0	2.40	16.0	2.77	10.0	2.30

petersii	2.25	0.81	8.0	2.08	9.0	2.20	27.0	3.30	10.0	2.30
peucephilus	1.40	0.34	5.0	1.61	6.7	1.90	18.0	2.89	6.0	1.79
philopunctatus	2.20	0.79	8.0	2.08	13.0	2.56	26.0	3.26	11.0	2.40
pigmaequestris	1.75	0.56	7.0	1.95	6.0	1.79	41.0	3.71	6.0	1.79
pinchoti	1.96	0.67	7.0	1.95	5.0	1.61	19.0	2.94	11.0	2.40
placidus	1.02	0.02	8.0	2.08	8.0	2.08	16.0	2.77	6.0	1.79
planiceps	2.26	0.82	5.0	1.61	8.0	2.08	16.0	2.77	9.5	2.25
poecilopus	1.62	0.48	7.0	1.95	7.0	1.95	18.0	2.89	19.0	2.94
pogus	2.56	0.94	4.0	1.39	6.0	1.79	19.0	2.94	7.0	1.95
polylepis	2.04	0.71	10.0	2.30	9.0	2.20	15.0	2.71	12.0	2.48
poncensis	2.61	0.96	3.0	1.10	3.0	1.10	17.0	2.83	6.0	1.79
porcatus	1.88	0.63	7.0	1.95	7.0	1.95	28.0	3.33	6.0	1.79
porcus	0.97	-0.03	12.0	2.48	3.0	1.10	31.0	3.43	8.0	2.08
princeps	2.24	0.81	9.0	2.20	9.0	2.20	23.0	3.14	15.0	2.71
proboscis	1.30	0.26	7.0	1.95	11.0	2.40	20.0	3.00	9.0	2.20
properus	1.30	0.26	8.0	2.08	11.0	2.40	18.0	2.89	4.0	1.39
pseudokemptoni	1.75	0.56	9.0	2.20	11.0	2.40	16.0	2.77	10.0	2.30
pulchellus	2.63	0.97	5.0	1.61	8.0	2.08	18.0	2.89	6.0	1.79
pumilus	1.50	0.41	4.0	1.39	11.0	2.40	17.0	2.83	6.0	1.79
punctatus	2.31	0.84	8.0	2.08	13.0	2.56	26.0	3.26	11.0	2.40
purpurgularis	2.18	0.78	5.0	1.61	6.0	1.79	16.0	2.77	9.0	2.20
pygmaeus	1.27	0.24	6.0	1.79	4.0	1.39	12.0	2.48	8.0	2.08
quadriocellifer	1.33	0.29	6.0	1.79	10.0	2.30	17.0	2.83	7.0	1.95
quaggulus	1.71	0.54	5.0	1.61	4.0	1.39	13.5	2.60	8.0	2.08
quercorum	1.83	0.60	5.0	1.61	6.0	1.79	13.0	2.56	7.0	1.95
ravitergum	1.44	0.36	8.0	2.08	11.0	2.40	20.0	3.00	4.0	1.39
reconditus	2.06	0.72	8.0	2.08	10.0	2.30	23.0	3.14	8.0	2.08
rejectus	2.70	0.99	4.0	1.39	4.0	1.39	15.0	2.71	8.0	2.08
richardi	1.80	0.59	8.0	2.08	11.0	2.40	25.0	3.22	8.0	2.08

ricordii	1.75	0.56	7.0	1.95	6.0	1.79	33.0	3.50	7.0	1.95
rimarum	2.41	0.88	7.0	1.95	16.0	2.77	20.0	3.00	9.0	2.20
rivalis	1.78	0.58	6.0	1.79	4.5	1.50	15.0	2.71	17.0	2.83
roatanensis	2.10	0.74	5.0	1.61	8.0	2.08	18.0	2.89	8.0	2.08
rodriguezii	2.00	0.69	7.0	1.95	15.0	2.71	17.0	2.83	9.0	2.20
roosevelti	1.50	0.41	7.0	1.95	6.0	1.79	35.0	3.56	7.0	1.95
roquet	1.70	0.53	7.0	1.95	10.0	2.30	26.0	3.26	9.0	2.20
rubiginosus	1.71	0.54	5.0	1.61	7.0	1.95	19.0	2.94	8.0	2.08
rubribarbaris	2.75	1.01	3.5	1.25	4.5	1.50	17.0	2.83	6.0	1.79
rubribarbus	1.50	0.41	7.0	1.95	11.0	2.40	17.0	2.83	8.0	2.08
ruizii	1.89	0.64	7.0	1.95	13.0	2.56	19.0	2.94	8.0	2.08
rupinae	2.10	0.74	6.0	1.79	9.0	2.20	19.0	2.94	12.0	2.48
sabanus	1.96	0.67	7.0	1.95	7.0	1.95	25.0	3.22	7.0	1.95
sagrei	2.08	0.73	6.0	1.79	7.0	1.95	19.0	2.94	7.0	1.95
salvini	1.31	0.27	7.0	1.95	10.0	2.30	18.0	2.89	6.0	1.79
schiedii	2.10	0.74	5.0	1.61	9.0	2.20	19.0	2.94	8.0	2.08
schwartzi	1.80	0.59	5.0	1.61	6.0	1.79	20.0	3.00	6.0	1.79
scriptus	1.38	0.32	6.0	1.79	9.0	2.20	22.0	3.09	6.0	1.79
scypheus	2.10	0.74	4.0	1.39	6.0	1.79	20.0	3.00	13.0	2.56
semilineatus	2.76	1.02	3.0	1.10	4.0	1.39	19.0	2.94	8.0	2.08
sericeus	2.00	0.69	4.5	1.50	7.0	1.95	15.0	2.71	7.0	1.95
serranoi	2.08	0.73	5.0	1.61	8.0	2.08	18.0	2.89	9.0	2.20
sheplani	1.13	0.12	9.0	2.20	11.0	2.40	17.0	2.83	6.0	1.79
shrevei	1.52	0.42	4.0	1.39	4.0	1.39	17.0	2.83	7.0	1.95
smallwoodi	2.03	0.71	6.0	1.79	6.0	1.79	34.0	3.53	6.0	1.79
smaragdinus	1.72	0.54	6.0	1.79	7.0	1.95	24.0	3.18	6.0	1.79
sminthus	2.37	0.86	4.0	1.39	4.0	1.39	18.0	2.89	6.0	1.79
soinii	2.68	0.99	8.0	2.08	11.0	2.40	19.0	2.94	12.0	2.48
solitarius	2.10	0.74	7.0	1.95	11.0	2.40	18.0	2.89	8.0	2.08

spectrum	2.30	0.83	4.0	1.39	3.0	1.10	15.0	2.71	6.0	1.79
squamulatus	2.33	0.85	10.0	2.30	13.0	2.56	24.0	3.18	13.0	2.56
strahmi	2.00	0.69	7.0	1.95	7.0	1.95	19.0	2.94	7.0	1.95
stratulus	1.50	0.41	8.0	2.08	11.0	2.40	20.0	3.00	7.0	1.95
subocularis	1.58	0.46	4.0	1.39	5.0	1.61	15.0	2.71	8.0	2.08
sulcifrons	1.63	0.49	8.0	2.08	9.0	2.20	20.0	3.00	9.0	2.20
tandai	1.92	0.65	4.0	1.39	7.0	1.95	14.0	2.64	12.5	2.53
taylori	1.75	0.56	8.0	2.08	9.0	2.20	19.0	2.94	7.0	1.95
terraealtae	1.45	0.37	7.0	1.95	7.0	1.95	26.0	3.26	6.0	1.79
terueli	1.96	0.67	4.0	1.39	10.0	2.30	17.0	2.83	4.0	1.39
tigrinus	1.59	0.46	8.0	2.08	9.0	2.20	19.0	2.94	7.0	1.95
tolimensis	2.15	0.77	7.0	1.95	9.0	2.20	17.0	2.83	9.0	2.20
townsendi	1.88	0.63	8.0	2.08	7.0	1.95	17.0	2.83	11.0	2.40
trachyderma	1.53	0.43	7.0	1.95	7.0	1.95	17.0	2.83	15.0	2.71
transversalis	2.02	0.70	10.0	2.30	17.0	2.83	24.0	3.18	7.0	1.95
trinitatis	1.51	0.41	9.0	2.20	12.0	2.48	25.0	3.22	8.0	2.08
tropidogaster	1.95	0.67	7.0	1.95	7.0	1.95	15.0	2.71	13.4	2.60
tropidolepis	1.52	0.42	6.0	1.79	8.5	2.14	14.0	2.64	14.0	2.64
tropidonotus	1.91	0.65	3.0	1.10	3.0	1.10	16.0	2.77	9.0	2.20
uniformis	1.55	0.44	3.0	1.10	3.0	1.10	14.0	2.64	8.0	2.08
unilobatus	1.93	0.66	5.0	1.61	8.0	2.08	13.0	2.56	8.0	2.08
utilensis	1.69	0.52	10.0	2.30	10.0	2.30	19.0	2.94	6.0	1.79
valencienni	1.31	0.27	13.0	2.56	8.0	2.08	26.0	3.26	6.0	1.79
vanidicus	1.92	0.65	4.0	1.39	3.0	1.10	15.0	2.71	6.0	1.79
vanzolinii	1.30	0.26	7.0	1.95	3.0	1.10	26.0	3.26	4.0	1.39
vaupesianus	2.24	0.81	7.0	1.95	13.0	2.56	24.0	3.18	10.0	2.30
ventrimaculatus	1.95	0.67	10.0	2.30	12.0	2.48	19.0	2.94	16.0	2.77
vermiculatus	2.11	0.75	9.5	2.25	10.5	2.35	29.0	3.37	10.0	2.30
villai	1.85	0.62	7.4	2.00	7.0	1.95	12.0	2.48	9.0	2.20

vittigerus	1.97	0.68	4.0	1.39	6.0	1.79	18.0	2.89	10.0	2.30
wattsi	1.94	0.66	4.0	1.39	5.0	1.61	19.0	2.94	6.0	1.79
websteri	1.41	0.34	6.0	1.79	11.0	2.40	18.0	2.89	6.0	1.79
wellbornae	2.10	0.74	5.0	1.61	7.0	1.95	14.0	2.64	7.5	2.01
whitemani	1.79	0.58	7.0	1.95	6.0	1.79	19.0	2.94	9.0	2.20
williamsmittermeierorun	1.36	0.31	7.0	1.95	8.0	2.08	18.0	2.89	5.0	1.61
woodi	2.30	0.83	7.0	1.95	8.0	2.08	17.0	2.83	12.0	2.48
yoroensis	1.69	0.52	5.0	1.61	7.0	1.95	14.0	2.64	11.0	2.40
zeus	2.06	0.72	6.5	1.87	9.0	2.20	17.0	2.83	10.0	2.30
