# Supplementary material for: The existence and evolution of morphotypes in Anolis lizards: coexistence patterns, not adaptive radiations, distinguish mainland and island faunas
Source: PeerJ. 2019 Jan 3;6:e6040. doi: 10.7717/peerj.6040 (PMC6321754; doi:10.7717/peerj.6040)
Supplement: Supplemental Information 2 [file peerj-07-6040-s002.pdf]

Sample	Island(0)/Mainland(1)	NTaxa	Number of morphotyes N=8	N=18	N=27	N=73
AguaOpisbo.MEX	1	3	2	2	3	3
Alfombra.CR	1	7	2	3	4	5
Andros.Island	0	4	2	3	4	4
Anegada	0	3	2	2	2	3
AssemblageI.CR	1	2	1	2	2	2
AssemblageII.CR	1	4	2	2	3	4
AssemblageIII.CR	1	7	2	3	4	5
AssemblageIV.CR	1	3	1	2	3	3
BajoCarara.Midsite.CararaNa	1	5	1	2	3	5
Baramita.GUY	1	2	1	2	2	2
Barbuda1	0	2	2	2	2	2
BarroColoradoIsland.PAN	1	10	3	5	7	8
Belem.BZ	1	3	1	1	2	2
BenitoJuarezSegundo.MEX	1	8	3	5	6	7
Berriozabal.MEX	1	7	2	4	5	6
Bijagual.HighSite.CararaNati	1	6	1	2	3	5
BilsaBiologicalStation.ECU	1	9	3	5	6	8
Boquete.PAN	1	10	3	4	5	5
BovoniCay1	0	2	2	2	2	2
Buenaventura.CO	1	9	3	5	6	8
CabezadePerro1	0	2	1	1	1	2
Caja.de.Muertos	0	3	1	1	1	2
Cali.km18.CO	1	5	3	3	3	3
CararaNationalPark.CR	1	7	1	2	3	6

CareeningIsland1	0	2	1	2	2	2
CaymanBrac1	0	2	2	2	2	2
Cayo.Alto	0	3	3	3	3	3
Cayo.Avalos	0	4	3	3	3	3
Cayo.Caguanes	0	7	3	6	6	7
Cayo.Campo	0	3	2	2	2	2
Cayo.Coco	0	7	3	5	6	7
Cayo.Conuco	0	4	3	3	3	3
Cayo.el.Rosario	0	3	3	3	3	3
Cayo.Ensenachos	0	4	3	4	4	4
Cayo.Fragoso	0	3	2	3	3	3
Cayo.Frances	0	6	3	5	5	5
Cayo.Guajaba	0	6	3	5	6	6
Cayo.Guillermo	0	4	2	3	4	4
Cayo.Ines.de.Soto	0	3	3	3	3	3
Cayo.Judas	0	3	3	3	3	3
Cayo.las.Brujas	0	6	3	5	6	6
Cayo.Lucas	0	3	2	3	3	3
Cayo.Palma	0	3	2	3	3	3
Cayo.Paredon.Grande	0	5	2	3	4	4
Cayo.Real	0	5	3	4	4	4
Cayo.Sabinal	0	7	3	5	6	7
Cayo.Saetia	0	8	4	5	6	7
Cayo.Salinas	0	4	2	4	4	4
Cayo.Santa.Maria	0	8	3	5	6	7
CayoAguado1	0	2	1	2	2	2

CayoBahiadeCadiz1	0	2	2	2	2	2
CayoCruzdelPadre1	0	2	2	2	2	2
CayoCueva1	0	2	2	2	2	2
CayoLanzanillo1	0	2	2	2	2	2
CayoLevisa1	0	2	2	2	2	2
Cayos.de.las.Cinco.Leguas	0	3	2	2	2	2
Cayos.los.Ballenatos	0	4	3	3	3	4
CayosCochinos.HON	0	2	2	2	2	2
CaySal1	0	2	2	2	2	2
CentralCaribbeanSlope.HON	1	6	3	5	5	6
CerroDantas.CR	1	3	1	2	2	2
CerroGuaiquinima.VEN	1	2	1	2	2	2
CerroGuanacaure.HON	1	2	1	2	2	2
Chinaja.AltaVarapaz.GUAT	1	6	2	4	4	4
Coapilla.MEX	1	2	1	2	2	2
CochaCashu.PER	1	4	1	2	3	3
Coscomatepac.MEX	1	2	1	1	2	2
CrookedIsland1	0	2	2	2	2	2
CuscoAmazonico.CA.PER	1	3	1	1	2	2
Cuyabeno.ECU	1	6	1	2	4	5
East-centralCaribbeanLowlan	1	9	2	5	7	7
EasternCaribbeanLowlands.H	1	10	2	5	7	8
EasternCaribbeanSlope.HON	1	6	2	4	5	5
EcoAmazonia.EA.PER	1	4	1	2	3	3
ECSA-RO.Jacupiranga.BZ	1	2	1	2	2	2
EHB-AM.Jacupiranga.BZ	1	3	1	2	3	3

ElQuebracho.NIC	1	4	2	3	4	4
ElYunque.PR	0	8	4	6	6	8
FlotaFaro.PER	1	2	1	2	2	2
Goat.Island	0	3	1	2	2	3
GrandPiedra.CBA	0	7	5	7	7	7
GraoParaSouthern.PER	1	2	1	1	2	2
GreatBirdIsland1	0	2	2	2	2	2
GreatCornIsland.NIC	0	2	1	2	2	2
GreatStirrupCay1	0	2	2	2	2	2
HaciendaBaru.Dominical.CR	1	7	1	2	4	6
HQ.lowsite.CararaNationalPar	1	4	1	2	3	4
IgarapéEsperaçanaReservaEx	1	6	1	2	4	5
Ile-a-Vache	0	3	2	3	3	3
Ile.a.Cabrit	0	5	3	3	3	3
Ile.de.la.Tortue	0	4	4	4	4	3
Ile.Grande.Cayemite	0	4	3	4	4	4
INPA-WWFReservenearManau	1	4	1	2	3	3
Iquitos.region.PER	1	7	1	2	4	5
Isla.Cabras	0	3	3	3	3	3
Isla.Catalina	0	3	3	3	3	3
Isla.de.la.Juventud	0	11	5	8	8	10
Isla.Saona	0	3	2	3	3	3
IslaBeata1	0	2	2	2	2	2
IslaMagueyes1	0	2	2	2	2	2
Jatapu.PER	1	3	1	2	2	3
Kartabo.GUY	1	5	1	2	3	4

Kingston.JAM	0	6	2	4	4	5
Konawaruk.GUY	1	2	1	2	2	2
LaBotija.HON	1	2	1	2	2	2
LaFlorida.CR	1	4	1	2	3	3
LaPalma.DR	0	7	3	6	7	7
LaPazWaterfall.CR	1	5	2	4	5	5
LaSelva.CR	1	8	2	3	5	6
LasPerlasIsland.PAN	0	3	2	3	3	3
LasVegas.Chiapas.MEX	1	3	1	3	3	3
LighthouseAtoll.BLZ	0	3	2	2	2	3
LittleCayman1	0	2	2	2	2	2
Long.Island	0	4	1	3	4	4
LowerMotaguaValley.HON	1	4	2	4	4	4
MadreSelva.PER	1	6	1	2	3	4
Manu.PER	1	3	1	2	3	3
Mindo.ECU	1	4	3	3	3	3
MocoronMultipleUseZone.HO[illegible]	1	5	1	3	3	3
MushaCay1	0	2	2	2	2	2
NassuaPlateau.SUR	1	3	1	2	2	2
North.Bimini	0	3	2	3	3	3
Ometepe.NIC	1	4	1	3	3	3
Omiltepe.MEX	1	3	2	2	2	3
ParqueNacionalLamura.HON	1	4	2	3	4	4
ParqueNacionalPicoBonito.Hi[illegible]	1	2	2	2	2	2
ParqueNacionalPicoBonito.Lo[illegible]	1	3	2	3	3	3
peucephilustype-locality.MEX	1	2	2	2	2	2

PinotepaNacional.MEX	1	3	1	1	1	2
Pochutla.MEX	1	3	2	2	2	3
QuebradaShinganatza.ECU	1	2	1	1	1	2
RafikiLodge.RioSavegre.CR	1	4	1	2	3	4
RebioMaicuru.PER	1	3	1	2	2	2
RincondeOsa.FundacionNeotr	1	7	1	2	4	5
RioKrutaBiologicalReserve.H(	1	4	1	3	4	4
RioPurus.Amazonia.BZ	1	5	1	2	3	4
RioSanJuan.NIC	1	8	2	4	5	6
RioWarunta.HON	1	6	2	3	4	6
RusRusBiologicalReserve.HOI	1	7	2	4	5	5
SanCristobal.MEX	1	2	2	2	2	2
SanIsidrodeDota.ReservaFore	1	6	1	2	4	5
SantaBarbaraHighlands.HON	1	2	2	2	2	2
SantaCecilia.ECU	1	6	1	2	4	5
SantaRosaNP.CR	1	3	1	2	3	3
Serraniagua.CO	1	7	4	4	4	4
ShipsternNatureReserve.BLZ	1	5	1	3	4	4
SierradeTrinidad.CBA	0	6	3	6	6	6
SierraNevadadeSantaMarta.(	1	3	2	3	3	3
Soroa,CBA	0	11	4	9	10	10
St.Bart1	0	2	1	1	2	2
St.Kitts1	0	2	2	2	2	2
St.Vincent	0	3	2	3	3	3
SugarLoaf1	0	2	2	2	2	2
Tapanti.CR	1	5	3	4	5	5

Tenorio.CR	1	6	1	3	4	5
Terán.Yacopi.CO	1	2	1	2	2	2
TierraColorado.MEX	1	3	1	2	2	3
Tortola	0	4	3	3	3	4
UHLEM-TO.Jacupiranga.BZ	1	2	1	2	2	2
UtilaIsland.HON	0	3	2	3	3	3
VolcanPoas.CR	1	3	2	3	3	3
Water.Cay	0	3	2	2	3	3
West-centralCaribbeanlowlan	1	7	3	5	6	6
WesternCaribbeanLowlands.H	1	8	2	4	5	6
WesternCaribbeanSlope.HON	1	5	2	3	3	4
WestPlana1	0	2	2	2	2	2
YojoaUplands.HON	1	3	2	3	3	3
