# Supplementary material for: The existence and evolution of morphotypes in Anolis lizards: coexistence patterns, not adaptive radiations, distinguish mainland and island faunas
Source: PeerJ. 2019 Jan 3;6:e6040. doi: 10.7717/peerj.6040 (PMC6321754; doi:10.7717/peerj.6040)
Supplement: Supplemental Information 3 [file peerj-07-6040-s003.pdf]

	Morphotype			
	N=8	N=18	N=27	N=73
acutus	1	1	1	1
aeneus	1	1	1	6
aequatorialis	4	12	19	56
agassizi	3	8	15	43
agueroi	7	17	26	71
ahli	1	1	1	3
aliniger	1	1	1	1
allisoni	3	9	16	48
allogus	1	1	1	3
altae	1	1	2	7
altavelensis	2	6	10	31
altitudinalis	2	6	11	34
alumina	1	2	3	11
alutaceus	1	4	8	29
alvarezdelto	1	1	1	3
amplisquam	5	14	22	62
anchicayae	1	3	5	21
anfiloquiae	1	4	8	29
angusticeps	2	7	13	39
annectens	1	5	9	30
antioquiae	4	12	19	56
antonii	1	1	2	7
apletophallu	1	1	2	8
apollinaris	3	8	15	44
aquaticus	1	3	5	25
argenteolus	1	1	1	4
argillaceus	2	6	12	36
armouri	1	2	3	12
auratus	5	14	23	65
bahorucoens	1	2	4	17
baleatus	3	10	17	52
baracoae	3	10	17	52
barahonae	3	10	17	52
barbatus	7	17	26	71
barbouri	5	14	23	68
barkeri	3	8	15	46

bartschi	1	1	1	4
beckeri	2	6	10	32
bellipeniculu	2	6	11	34
benedikti	1	3	5	23
bicaorum	1	3	6	26
bimaculatus	3	9	16	50
binotatus	1	3	5	24
biporcatus	1	3	6	26
biscutiger	1	1	2	8
blanquillanus	3	9	16	49
bocourti	1	1	2	7
boettgeri	1	1	2	7
bombiceps	1	3	6	27
bonairensis	1	1	1	1
boulengeriar	1	2	3	13
brasiliensis	1	2	3	12
bremeri	1	2	4	19
breslini	1	2	4	20
brevirostris	2	6	10	31
brunneus	2	6	11	35
calimae	1	1	2	7
campbelli	1	2	3	16
capito	1	3	6	26
caquetae	1	1	1	4
carlostoddi	2	6	11	34
carolinensis	3	9	16	49
carpenteri	1	1	2	9
casildae	4	12	19	58
caudalis	2	6	10	31
centralis	2	6	12	36
chamaeleoni	7	17	26	71
charlesmyeri	1	1	1	6
chloris	1	1	2	7
chlorocyanus	3	9	16	49
chocorum	4	12	19	57
christophei	1	3	5	21
chrysolepis	1	3	6	27
chrysops	3	9	16	50

clivicola	1	4	8	29
cobanensis	1	3	5	21
coelestinus	1	1	1	2
compressica	1	2	3	13
concolor	1	2	4	19
confusus	2	6	10	33
conspersus	1	1	1	4
cooki	1	2	4	19
crassulus	5	14	23	65
cristatellus	1	2	4	19
cristifer	1	1	1	6
cryptolimifrc	1	1	2	8
cupeyalensis	5	14	22	64
cupreus	1	3	5	24
cuprinus	1	3	5	25
cuscoensis	1	1	2	7
cusuco	1	2	3	14
cuvieri	3	10	17	53
cybotes	1	2	4	19
danieli	3	8	15	44
datzorum	2	6	10	31
desechensis	1	2	4	20
desiradei	3	9	16	49
dissimilis	2	6	10	32
distichus	2	6	10	31
dolichocepha	1	2	4	17
dollfusianus	1	3	5	24
dominicensis	2	6	10	33
duellmani	5	15	24	69
dunni	1	2	4	20
equestris	3	10	17	52
ernestwillian	3	9	16	49
etheridgei	1	1	2	8
eugenegraha	4	12	19	56
eulaemus	4	12	19	56
evermanni	3	9	16	49
extremus	1	1	1	2
fairchildi	3	9	16	49

fasciatus	4	12	19	57
favillarum	2	6	10	33
ferreus	3	9	16	51
festae	1	1	2	7
fitchi	4	12	19	56
forresti	1	2	3	15
fortunensis	1	1	2	7
fowleri	3	8	15	47
fraseri	3	8	15	47
frenatus	3	8	15	45
fugitivus	5	14	22	64
fungosus	2	7	13	40
fuscoauratu:	1	1	2	7
gadovii	1	3	6	26
gaigei	1	3	5	24
garmani	3	10	17	53
garridoi	2	7	13	40
gemmosus	4	12	19	56
ginaelisae	3	8	15	47
gingivinus	1	2	4	19
gorgonae	1	1	2	7
gracilipes	1	3	5	23
grahami	1	1	1	2
granuliceps	4	13	21	60
griseus	3	8	15	43
gruuo	1	1	2	7
guamuhaya	7	17	26	71
guazuma	2	7	13	40
gundlachi	1	3	6	26
haetianus	1	2	4	19
hendersoni	1	2	4	17
heterodermı	8	18	27	72
heteropholio	1	2	3	15
hobartsmith.	1	1	2	8
homolechis	1	2	4	19
huilae	3	8	15	47
humilis	5	15	24	69
ibanezi	4	12	19	57

ignigularis	2	6	10	33
imias	1	1	1	3
inexpectatus	1	4	8	29
insignis	3	8	15	44
insolitus	2	7	14	42
isolepis	2	6	11	34
jacare	1	1	1	1
johnmeyeri	1	2	3	12
juangundlach	5	14	22	64
jubar	2	6	10	31
kahouannens	3	9	16	49
kemptoni	1	1	2	7
koopmani	5	14	23	67
krugi	1	2	4	20
kunayalae	4	12	20	59
laeviventris	1	2	3	14
latifrons	4	12	19	58
leachii	3	9	16	50
lemurinus	1	3	6	26
limifrons	1	1	2	8
lineatopus	1	1	1	2
lineatus	1	2	4	19
liogaster	1	2	3	13
lionotus	1	3	5	25
litoralis	2	6	12	36
lividus	3	9	16	49
longiceps	3	9	16	49
longitibialis	1	2	3	12
loveridgei	3	8	15	45
loysianus	2	6	12	36
luciae	1	1	1	2
lucius	1	1	1	4
luteogularis	3	10	17	54
luteosignifer	2	6	10	31
lynchi	4	13	21	61
lyra	1	3	6	26
macilentus	1	4	8	29
macrinii	3	8	15	47

macrolepis	1	3	5	25
macrophallus	1	2	3	12
maculigula	4	12	19	58
maculiventri	1	1	2	8
magnaphallu	1	3	5	24
marcanoi	1	2	3	12
mariarum	1	1	2	7
marmoratus	3	9	16	49
marron	2	6	10	31
marsupialis	5	15	24	69
matudai	1	2	3	12
maynardi	3	9	16	49
medemi	1	1	2	7
megalopithe	4	12	19	56
megapholido	5	14	23	66
menta	2	6	10	32
meridionalis	1	5	9	30
microlepidot	2	6	12	36
microtus	3	8	15	47
milleri	1	3	5	22
monensis	1	1	1	3
monteverde	2	6	10	31
monticola	1	3	5	21
morazani	5	14	22	63
naufragus	1	3	5	22
neblininus	2	6	11	34
nebuloides	1	2	3	13
nebulosus	1	2	3	14
nelsoni	1	2	4	18
nicefori	2	6	11	34
noblei	3	10	17	52
notopholis	5	14	23	67
nubilus	3	9	16	49
occultus	6	16	25	70
ocelloscapul	1	1	2	8
oculatus	3	9	16	50
oligaspis	2	7	13	39
olssoni	5	14	22	63

omiltemanus	1	2	3	14
onca	1	3	6	26
opalinus	1	1	1	1
ophiolepis	5	14	23	67
orcesi	2	7	14	42
ortonii	1	1	2	7
oxylophus	1	3	5	25
pachypus	1	3	5	23
parilis	4	12	20	59
parvauritus	1	3	6	26
parvicirculat	1	3	5	21
paternus	2	7	13	40
pentaprion	1	1	1	6
peraccae	1	1	2	7
petersii	3	8	15	44
peucephilus	2	6	12	36
philopunctat	1	1	1	5
pigmaeques	3	10	17	52
pinchoti	1	3	5	21
placidus	2	7	13	41
planiceps	1	3	6	26
poecilopus	1	3	5	23
pogus	1	2	3	11
polylepis	1	1	2	7
poncensis	5	14	22	63
porcatus	3	9	16	49
porcus	7	17	26	71
princeps	4	12	19	58
proboscis	1	1	1	6
properus	2	6	10	33
pseudokemp	1	1	2	7
pulchellus	1	2	4	17
pumilus	2	6	12	37
punctatus	1	1	1	5
purpurgularis	1	2	3	12
pygmaeus	5	15	24	69
quadriocellif	2	6	10	31
quaggulus	5	15	24	69

quercorum	1	2	3	14
ravitergum	2	6	10	33
reconditus	3	8	15	47
rejectus	5	14	22	64
richardi	3	8	15	43
ricordii	3	10	17	52
rimarum	1	1	2	10
rivalis	1	3	7	28
roatanensis	1	2	4	18
rodriguezii	1	1	2	10
roosevelti	3	10	17	52
roquet	1	1	1	2
rubiginosus	1	2	4	20
rubribarbaris	5	14	22	63
rubribarbus	1	1	1	3
ruizii	1	1	2	7
rupinae	1	1	2	8
sabanus	3	9	16	49
sagrei	1	2	4	19
salvini	2	6	10	31
schiedii	1	2	4	18
schwartzi	1	2	4	20
scriptus	3	9	16	49
scypheus	1	3	6	27
semilineatus	5	14	22	63
sericeus	1	2	3	14
serranoi	1	3	6	26
sheplani	2	7	13	41
shrevei	1	2	3	13
smallwoodi	3	10	17	52
smaragdinus	2	6	11	34
sminthus	5	14	22	63
soinii	4	12	19	57
solitarius	1	1	2	7
spectrum	5	14	22	62
squamulatus	4	12	19	56
strahmi	1	2	4	19
stratulus	2	6	10	31

subocularis	1	2	3	13
sulcifrons	1	1	1	3
tandai	1	3	6	27
taylori	1	1	1	3
terraealtae	3	9	16	49
terueli	2	6	12	38
tigrinus	2	6	10	32
tolimensis	1	1	2	7
townsendi	1	3	5	21
trachyderma	1	3	5	23
transversalis	1	1	1	4
trinitatis	1	1	1	6
tropidogaste	1	3	5	23
tropidolepis	1	3	5	23
tropidonotus	5	14	23	65
uniformis	5	14	23	66
unilobatus	1	2	3	14
utilensis	2	6	10	32
valencienni	3	11	18	55
vanidicus	5	14	22	62
vanzolinii	8	18	27	73
vaupesianus	1	1	1	5
ventrimacula	4	12	19	56
vermiculatus	3	8	15	44
villai	1	3	5	22
vittigerus	1	2	3	12
wattsi	1	2	3	15
websteri	2	6	10	31
wellbornae	1	2	3	14
whitemani	1	3	5	21
williamsmitte	2	6	11	34
woodi	1	3	6	26
yoroensis	1	3	5	24
zeus	1	1	2	8
