# Supplementary material for: The existence and evolution of morphotypes in Anolis lizards: coexistence patterns, not adaptive radiations, distinguish mainland and island faunas
Source: PeerJ. 2019 Jan 3;6:e6040. doi: 10.7717/peerj.6040 (PMC6321754; doi:10.7717/peerj.6040)
Supplement: Supplemental Information 4 — Ranges (e.g., “1–2”) represent variation across 100 phylogenetic estimates. Numbers refer to morphotypes listed in Table S3 of the Supplemental Information. [file peerj-07-6040-s004.docx]

| Morphotype | No. changes to morphotype within islands | No. changes to morphotype within mainland |
| --- | --- | --- |
| 1 | 4 | 1 |
| 2 | 5 | 0 |
| 3 | 2 | 3 |
| 4 | 3 | 2 |
| 5 | 0 | 1 |
| 6 | 3 | 3 |
| 7 | 0 | 8-10 |
| 8 | 2 | 4-5 |
| 9 | 0 | 1 |
| 10 | 1 | 1 |
| 11 | 1 | 1 |
| 12 | 3 | 5-6 |
| 13 | 1 | 4-5 |
| 14 | 0 | 4-6 |
| 15 | 1-2 | 1 |
| 16 | 0 | 1 |
| 17 | 2 | 0 |
| 18 | 2 | 1 |
| 19 | 10-11 | 0 |
| 20 | 4 | 2 |
| 21 | 5 | 3 |
| 22 | 1 | 2 |
| 23 | 0 | 5-6 |
| 24 | 0 | 5-6 |
| 25 | 0 | 4-5 |
| 26 | 1 | 7-8 |
| 27 | 0 | 1-2 |
| 28 | 0 | 1 |
| 29 | 3-4 | 0 |
| 30 | 0 | 1-2 |
| 31 | 6 | 2-3 |
| 32 | 0 | 2-4 |
| 33 | 2 | 0 |
| 34 | 2-3 | 2-4 |
| 35 | 1-2 | 0 |
| 36 | 1-3 | 2 |
| 37 | 1 | 0 |
| 38 | 1 | 0 |
| 39 | 1 | 0 |
| 40 | 1-2 | 1 |
| 41 | 1 | 0 |
| 42 | 1 | 1 |
| 43 | 2-3 | 0 |
| 44 | 1 | 4 |
| 45 | 0 | 2 |
| 46 | 0 | 1 |
| 47 | 2 | 4 |
| 48 | 1 | 0 |
| 49 | 9-12 | 0 |
| 50 | 1-4 | 0 |
| 51 | 1 | 0 |
| 52 | 2 | 0 |
| 53 | 2 | 0 |
| 54 | 1 | 0 |
| 55 | 1 | 0 |
| 56 | 1 | 2 |
| 57 | 0 | 3-4 |
| 58 | 0 | 1-3 |
| 59 | 0 | 1-2 |
| 60 | 0 | 1 |
| 61 | 0 | 1 |
| 62 | 1 | 1 |
| 63 | 2-3 | 2 |
| 64 | 1-3 | 0 |
| 65 | 0 | 3 |
| 66 | 0 | 2 |
| 67 | 2 | 1 |
| 68 | 1 | 0 |
| 69 | 0 | 3-4 |
| 70 | 1 | 0 |
| 71 | 1 | 0 |
| 72 | 0 | 1 |
| 73 | 0 | 1 |
